# EV-B 3D polymerase remodels viral populations through 5′UTR recombination to subvert cardiac antiviral innate immunity

**DOI:** 10.1371/journal.ppat.1014441

**Published:** 2026-07-24

**Authors:** Marie Glenet, Laetitia Heng, Anne-Laure Lebreil, Paul-Antoine Gretteau, Yohan Nguyen, Fatma Berri, Laurent Andreoletti

**Affiliations:** 1 Inserm, UMR-S1320 CardioVir, University of Reims Champagne Ardennes, Reims, France; 2 Infectious Diseases Department, Academic Hospital of Reims, Robert Debré, Reims, France; 3 Medical Virology Department, Academic Hospital of Reims, Robert Debré, Reims, France; University of Maryland at College Park: University of Maryland, UNITED STATES OF AMERICA

## Abstract

Viral myocarditis, a leading cause of morbidity in young populations, is strongly linked to Coxsackievirus B (CV-B) infections harboring dominant 5′-terminally deleted (5′TD) and minor full-length (FL) CV-B RNA populations in cardiac tissues. Here, we demonstrate how viral RNA-dependent RNA polymerase (3Dpol)-driven recombination in the 5′UTR orchestrates viral RNA populations dynamics and subverts type I interferon responses. In primary human cardiomyocytes (HCMs), 3Dpol-mediated copy-choice recombination enhances 5′TD RNA replication while suppressing FL populations. Infection of immunocompetent mice with recombination-deficient CV-B3 (3Dpol Y276H) shifted 5′TD populations ratios toward immune-sensing viral RNAs, elevating cardiac IFN-β/ISG15 and accelerating viral clearance. Transfection experiments confirmed that 50-nt 5′TD RNAs (TD50) evade innate immunity, whereas shorter deletions (9–36-nt, TD15) restore type I interferon responses in HCMs. Our findings establish 3Dpol-driven recombination as a critical mechanism sustaining pathogenic 5′TD RNAs that subvert antiviral innate immunity, highlighting recombination inhibition as a promising therapeutic strategy against CV-B myocarditis.

## Author summary

Viral myocarditis is an inflammation of the heart muscle that can cause heart failure in children and young adults. Coxsackievirus B is a major viral cause of this disease and can sometimes persist in the heart for months or years. In infected hearts, the virus often exists as a mixture of “complete” genomes and shorter genomes missing a segment at their beginning. Until now, it was unclear how these shortened genomes are maintained and how they help the virus evade the immune system. This study demonstrates that a viral enzyme called 3D polymerase, which copies the viral genetic material, can jump from one RNA strand to another during copying, a process known as copy-choice recombination. This process creates and enriches shortened genomes that are more difficult for the body’s antiviral defenses to detect. When this recombination process is blocked in heart cells and in mice, the immune system recognizes the virus more effectively and clears it more rapidly. These findings suggest that specifically targeting viral recombination could lead to future antiviral therapies for Coxsackievirus-induced myocarditis.

## Introduction

Enteroviruses B (EV-B) belong to the genus of enteroviruses from the *Picornaviridae* family. They are small, naked, single-stranded, positive RNA viruses with about 7,500 nucleotides. The coding sequence is flanked at both ends by untranslated regions (UTR) [1–3]. The 5′-UTR consists of two secondary structural complexes that are crucial for the initiation of replication and translation of the viral genome. The first is a cloverleaf-like structure involved in RNA replication and consists of a stem “a” and three stem loops “b”, “c”, and “d” [1–3]. The second structure is an internal ribosome entry site (IRES) composed of six stem loops (SL), which is involved in the translation of the viral polyprotein [4].

Enterovirus B (EV-B) species are ubiquitous viruses transmitted primarily through fecal-oral or respiratory routes. While most EV-B infections in humans are asymptomatic, coxsackieviruses of group B (CV-B) are notable pathogens responsible for acute infections, including meningitis, pancreatitis, and myocarditis [5–7]. In some patients, acute CV-B infections can progress to persistent infections, leading to chronic pathologies. For instance, 10–20% of myocarditis cases evolve into chronic myocarditis, which may result in dilated cardiomyopathy (DCM), progressing to chronic cardiac failure and ultimately necessitating heart transplantation [8].

A growing body of evidence highlights the presence of EV-B populations with 5′-terminal deletions (EV-B 5′TD) in patients with acute or chronic infections. These populations are found alongside a minor proportion (~5%) of full-length (FL) RNA forms [9–11]. Similar observations have been made in an immunocompetent mouse model of acute and persistent cardiac infection induced by a cardiotropic CV-B3 strain [12,13]. Notably, recent studies have demonstrated that early-emerging CV-B3 populations with deletions in domain I of the 5′-non-coding region (S1 Fig), corresponding to the cloverleaf RNA structure at the 5′end of the genomic RNA, modulate the type I interferon (IFN) signaling pathway in human cardiomyocytes and contribute to myocarditis in mice. These findings underscore the role of replicative CV-B 5′TD RNA forms as key pathophysiological factors in CV-B-induced human myocarditis [1,11].

The deleted viral populations are characterized by 5′-terminal deletions ranging from 7 to 50 nucleotides, which disrupt the cloverleaf structure and impair its functionality [3,14]. Deletions of the first 50 nucleotides are particularly predominant, characterizing the major viral RNA populations found in both human and murine cardiac samples [10,11,13,15]. These deletions remove a binding site for the cellular factor PCBP2 (poly (rC)-binding protein 2), located in stem-loop b [3,14], resulting in reduced genomic replication capacity [14]. In agreement with this observation, Hunziker et al. demonstrated that deletion of a 32-nt segment within the 5′UTR of the Woodruff strain completely abolished RNA replication in cells [16]. Notably, the corresponding sequence is highly conserved in the CV-B3/28 strain, further supporting the essential role of this region in viral RNA replication. However, the protease activities of these deleted forms remain intact, enabling them to impair cellular and tissue functionality [10,17].

Collectively, these studies suggest that EV-B 5′TD forms are generated and selected early during the natural course of infection. The emergence of these populations, despite their low replicative capacity, may play a critical role in the development of acute and chronic pathologies. However, the molecular mechanisms underlying the maintenance of these deleted forms in high proportions, alongside minor FL forms, remain poorly understood [17]. Intriguingly, co-transfection experiments have shown that the presence of major EV-B 5′TD forms (95%) alongside minor FL forms (5%)-mimicking the proportions observed in patients-generates significantly higher amounts of infectious particles in primary human cardiomyocytes (HCM) compared to FL virus alone [10]. These findings strongly suggest the existence of molecular interactions between 5′TD and FL populations, potentially involving the exchange of viral proteins or genomic sequences.

Recombination is a common process in positive-sense RNA viruses, particularly enteroviruses, and occurs via two non-exclusive mechanisms [18]. The replicative model relies on the ability of viral RNA polymerase to switch templates during the synthesis of the negative RNA strand, while the non-replicative model involves self-ligation or rejoining of broken RNA molecules mediated by cellular ligases or spontaneous ligation [19,20]. Genomic recombination mechanisms in the 5′-UTR may facilitate interactions between EV-B 5′TD and FL populations. Studies have shown that co-transfection of defective EV RNA, which cannot independently produce infectious particles, can be rescued by recombination events, leading to the formation of recombinant viruses [21–25]. Most recombination assays (CRE-REP assays) have been developed based on this principle and allow the study of recombination mechanisms in the coding region for non-structural proteins [21–24]. Another recombination research system has been developed to study RNA recombination events occurring exclusively in the 5′UTR [25].

In this study, we define the molecular mechanisms governing genomic RNA recombination within the 5′ untranslated region (5′UTR) of group-B enteroviruses. To dissect this process, we engineered a recombination model in human primary cardiomyocytes (HCM) using two replication-defective CV-B genomes, the 5′partner with the 5′UTR of CV-B6/Shmitt and the 3′partner corresponding to TD50 CV-B3/28. This system leverages either wild-type CV-B or a recombinant-deficient mutant (CV-B3 3Dpol Y276H), which carries a point mutation in the viral RNA-dependent RNA polymerase (3Dpol) that disrupts the copy-choice recombination mechanism. Recombinant viral strains generated through this approach were systematically characterized for genetic structure, replication fitness in HCM, and cardiovirulent phenotype in immunocompetent mice. Using this framework, we interrogated how 3Dpol-dependent recombination in the 5′UTR shapes the equilibrium of pathogenic 5′-terminally deleted (5′TD) RNA populations and their capacity to evade the type I interferon (IFN-I) response during myocarditis progression. Our findings directly link viral recombination activity to immune evasion and cardiac acute and chronic CV-B infections, offering new insights into enterovirus pathogenesis.

## Results

### Homologous and non-homologous recombination events in the enterovirus 5′UTR shapes viral fitness in human cardiomyocytes

**Fig 1:**
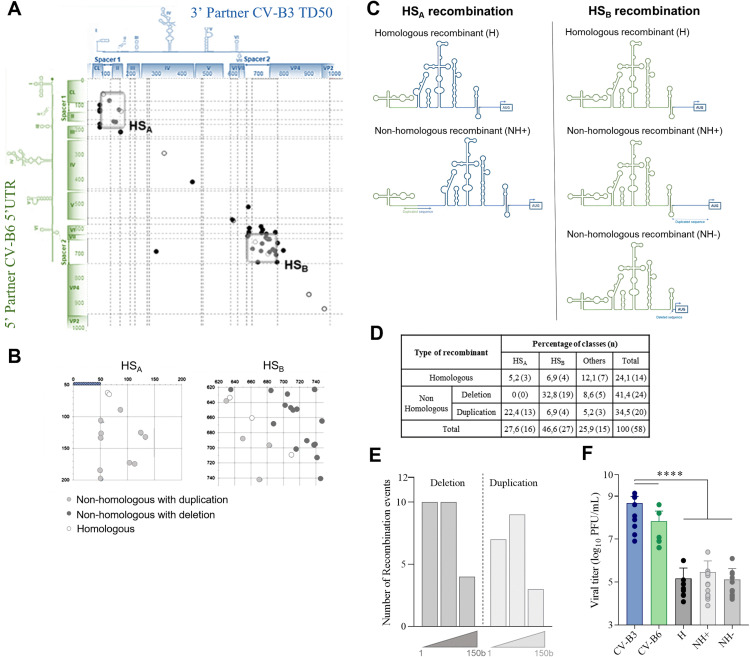
Characterization of genetic recombination events in the 5′ UTR of CV-B leading to infectious recombinants. **(A)** Recombination sites were mapped from purified infectious recombinants (*n* = 58) following co-transfection of the 3′ partner (3′P, X-axis) and the 5′ partner (5′P, Y-axis) in a 19:1 ratio in human cardiomyocytes (HCM). The locations of recombination events in the 5′ UTR are depicted, with spacers 1 and 2 identified as recombination hotspots, termed HS_A_ and HS_B_, respectively. **(B)** Hotspots HS_A_ and HS_B_ are highlighted, with recombination types depicted. **(C)** Representative recombinants of each type homologous (H), non-homologous with deletion (NH−), and non-homologous with insertion (NH+) are shown in HS_A_ and HS_B_. Sequences from the 5′ partner CV-B6 (green) and 3′ partner CV-B3 D50 (blue) are indicated. **(D)** The proportions and numbers of recombinant viruses are presented by recombination type and their position within the viral genome. **(E)** Distribution of NH recombinants according to the length (in bases) of the duplication or insertion. **(F)** Virus titers by recombinant type after 72 h of amplification in HCM. Data represent the mean ± SD. ****: *P* < 0.0001 by Mann–Whitney *U* test. HS: Hotspot, H: homologous, NH-: non-homologous with deletion, NH + : non-homologous with duplication.

The potential for genomic recombination mechanisms was evaluated between a major form of EV-B 5′TD RNA with a 49-nucleotide deletion (TD50) and a minor form of full-length (FL) RNA, as previously characterized [10,11]. We developed an experimental recombination model based on a previously established assay [25], designed to target recombination events specifically in the 5′ UTR by rescuing two defective viral genomes (S1 Fig). Following the co-transfection of both RNA partners, 58 recombinant viruses were isolated by plaque picking and their first 1000 nucleotides were sequenced (S1 Table). Recombination sites were identified by alignment with the parental partners. The recombination events are mainly concentrated in two regions considered as hotspots, spacer 1 (HS_A_) and 2 (HS_B_) with 27.6% and 46.6% events, respectively (*P* < 0.01, according to Fischer’s exact test) (Fig 1A and 1D). Sequencing revealed that 24.1% of the recombinants were homologous (H) and 75.9% were non-homologous (NH) recombinants characterized by nucleotide duplications (34.5%) (NH+) or nucleotide deletions (41.4%) (NH−) (Fig 1D). Deletions appeared to be the most common recombination events that occurred in the HS_B_ (32.8%), but none were identified in the HS_A_ (Fig 1B and 1D). Deletion and duplication went from 1 to a maximum of 147 and 135 nucleotides, respectively (Fig 1E). Viral titers of the isolated recombinant forms, ordered by recombination type after amplification, showed the same lower viral replication activity for each type of recombinant forms (*P* < 0.0001) compared with the parental forms (Fig 1F). Finally, our results showed that recombination events occurred mainly in two regions, producing recombinant strains with lower fitness than parental viruses.

**Fig 2:**
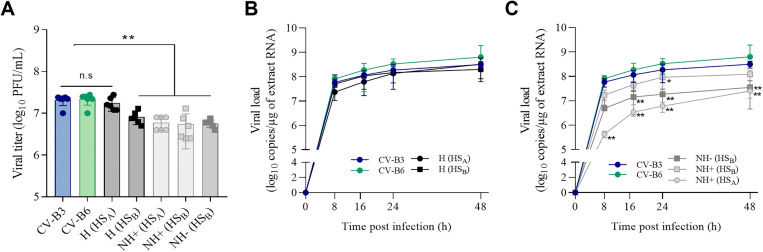
Fitness of CV-B recombinant strains compared to parental viruses. Human cardiomyocytes (HCM) were infected with a selected recombinant (H, NH^−^, or NH^+^) or with CV-B3 or CV-B6 parental viruses at an MOI of 10. **(A)** Viral titers of recombinant and parental viruses at 24 h post-infection. **(B)** Total viral RNA (intracellular and extracellular) from CV-B3 or CV-B6 parental viruses and homologous recombinants was measured by RT-qPCR at the indicated time points post-infection. **(C)** Total viral RNA (intracellular and extracellular) from CV-B3 or CV-B6 parental viruses and non-homologous recombinants was measured by RT-qPCR at the indicated time points post-infection. Data represent the mean ± SD for three independent experiments. *: *P* < 0.05; **: *P* < 0.01; The Mann–Whitney test was used for two groups comparison. CV-B: Coxsackievirus, H: homologous, HS: Hotspot, MOI: multiplicity of infection, NH: non-homologous, PFU: plage forming unit.

To assess the fitness of viral recombinants, one recombinant of each type was selected and evaluated for RNA replication activity in human cardiomyocytes (HCM). Viral infectious titers (PFU) demonstrated that recombinant viruses, particularly NH recombinants, produced fewer infectious particles compared to parental viruses (Fig 2A). Notably, the replication activity of homologous (H) recombinant strains, specifically HS_A_ was closer to that of parental strains than to homologous H (HS_B_) and NH recombinants. Moreover, the non-homologous (NH) recombinants exhibited significantly reduced RNA replication levels compared to the parental CV-B3 and CV-B6 strains, particularly at 24 HPI (*P* < 0.01 and *P* < 0.001, respectively) (Fig 2B and 2C).

### Copy-choice recombination mechanism is the predominant process driving recombination between viral RNA forms in human cardiomyocytes

**Fig 3:**
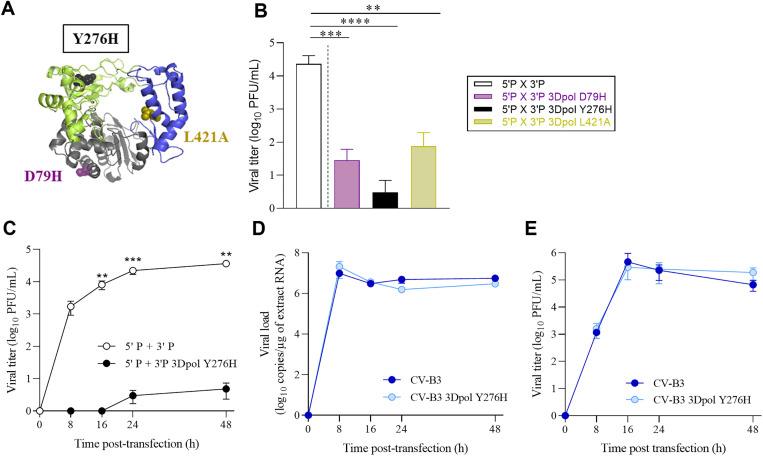
Assessment of 3D polymerase action on recombination mechanisms between 5′ and 3′ RNA partners. **(A)** Ribbon representation of CV-B3 3D polymerase, showing fingers subdomain (light green), thumb (dark blue), and palm (gray). Mutations impairing copy-choice recombination D79H, Y276H, and L421A are indicated. **(B)** Co-transfection of 5′ partner with either wild-type or mutant 3′ partners (3′P 3Dpol D79H, 3′P 3Dpol Y276H, or 3′P 3Dpol L421A) in a 19:1 ratio at 24 h post-transfection in HCM; infectious recombinant production was analyzed. **(C)** Co-transfection of 5′ partner with wild-type or mutant 3′ partner (3Dpol Y276H) at various time points post-transfection in HCM, with infectious recombinant production analysis. **(D, E)** Transfection of CV-B3 or CV-B3 Y276H mutant: (D) assessment of viral RNA replication activity and (E) analysis of infectious recombinant production. Data represent the mean ± SD for three independent experiments. **: P < 0.01; ***: P < 0.001; ****: P < 0.0001 by Mann Whitney *U* test. HCM: human cardiac myocytes, P: partner, 3Dpol: 3D polymerase, PFU: plaque forming unit.

Mutations in the viral polymerase that impair recombination mechanisms have been previously characterized in poliovirus and EV-A71 [22,26,27]. To investigate the recombination mechanism in our system, we engineered orthologous substitutions-D79H, Y276H, and L421A-into the 3D polymerase of the 3′ partner (Fig 3A).

Co-transfection of the 5′ partner with each of the three mutated 3′ partners resulted in a significant decrease in infectious recombinant levels compared to the non-mutated 3′ partner (Fig 3B). Among the mutations, 3′Dpol Y276H mutation had the most pronounced effect, severely impairing recombination events (Fig 3B and 3C) without affecting RNA replication activity or infectious particle production levels (Fig 3D and 3E). These findings demonstrate that the copy-choice recombination mechanism is the predominant process driving recombination between the 5′ and 3′ partners in HCM.

### 3Dpol-mediated copy-choice recombination enhances 5′ terminally deleted RNA forms replication while suppressing FL RNA genome populations

Previous work by our team demonstrated that co-transfection of 5′-terminally deleted (5′TD) and full-length (FL) CV-B3/28 RNA forms in human cardiomyocytes (HCM) induced higher viral titers compared to transfection of either form alone [10]. These findings, combined with our current results (Fig 3), suggest that 5′TD forms may interact with the 5′ UTR sequence of FL forms through copy-choice recombination, leading to positive or negative modulation of FL or 5′TD RNA replication activities.

**Fig 4:**
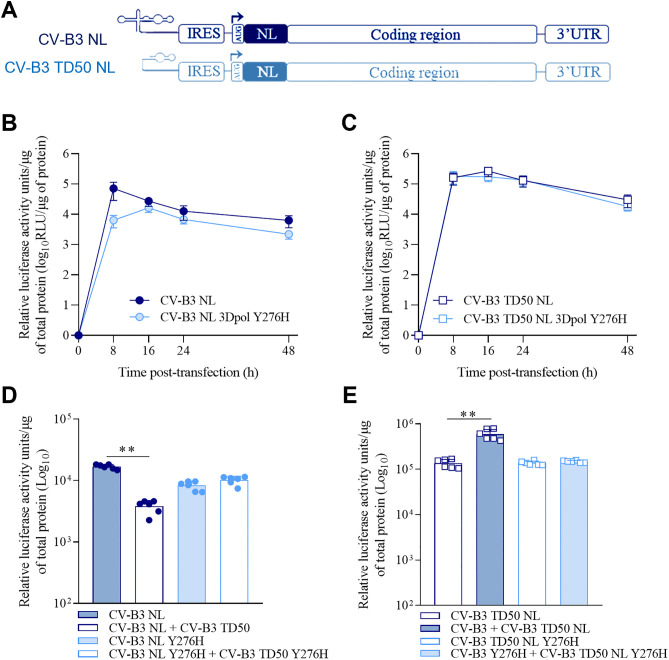
Impact of interaction between full-length and deleted CV-B3 RNA forms on viral translation activities. **(A)** Schematic representation of full-length (FL) and 5′ terminally deleted (TD50, D50) CV-B3 nanoluciferase-tagged (NL) RNAs. **(B)** Relative luciferase activity in human cardiomyocytes (HCM) following transfection with CV-B3 NL-tagged RNA, with or without the Y276H mutation in 3Dpol. **(C)** Relative luciferase activity in HCM after transfection with TD50 CV-B3 NL-tagged RNA, with or without Y276H mutation in 3Dpol. **(D)** Relative luciferase activity, normalized to total protein, following transfection of 5% FL CV-B3 NL-tagged RNA (with or without Y276H mutation in 3Dpol), alone or co-transfected with 95% TD50 CV-B3 RNA (with or without Y276H mutation). **(E)** Relative luciferase activity, normalized to total protein, following transfection of 95% TD50 CV-B3 NL-tagged RNA (with or without Y276H mutation in 3Dpol), alone or co-transfected with 5% FL CV-B3 RNA (with or without Y276H mutation). Data represent the mean ± SD for three independent experiments *: *P* < 0.05; ****: *P* < 0.0001 by Mann–Whitney *U* test. 3Dpol: 3D polymerase, CV-B: Coxsackievirus, IRES: Internal Ribosome Entry Site, NL: Nanoluciferase, TD: 5′terminally deleted, UTR: Untranslated region.

To investigate these intra-genomic interactions, we tagged 5′TD and FL CV-B3 RNA forms with nanoluciferase (NL), generating TD50 CV-B3 NL and CV-B3 NL, respectively (Fig 4A).

Introduction of the Y276H mutation in the 3Dpol of Nanoluciferase-tagged viruses did not affect translation activity levels at 24 h post-transfection compared to non-mutated Nanoluciferase-tagged RNA forms (Fig 4B and 4C). Co-transfection of 5′TD CV-B3 and FL CV-B3 NL resulted in significantly lower luciferase activity compared to FL CV-B3 NL alone (*P* < 0.01) (Fig 4D), indicating that 5′TD forms negatively modulate the replication fitness of FL forms in HCM. Conversely, co-transfection of 5% FL CV-B3 and 95% 5′TD CV-B3 NL showed significantly higher luciferase activity compared to 5′TD CV-B3 NL alone (*P* < 0.01) (Fig 4E), demonstrating a positive effect of FL forms on the replication of 5′TD RNA forms. When the 3′Dpol Y276H mutation was introduced into the RNA genome sequences, luciferase activities were indistinguishable between the RNA mixtures and the individual RNA forms (Fig 4D and 4E). Collectively, these findings demonstrate that copy-choice recombination events in cultured HCM negatively modulate the replication activity of FL RNA forms while enhancing the replication activity of 5′TD RNA forms.

### 3D-pol mediated recombination activity controls the natural course of EV cardiac infection

**Fig 5:**
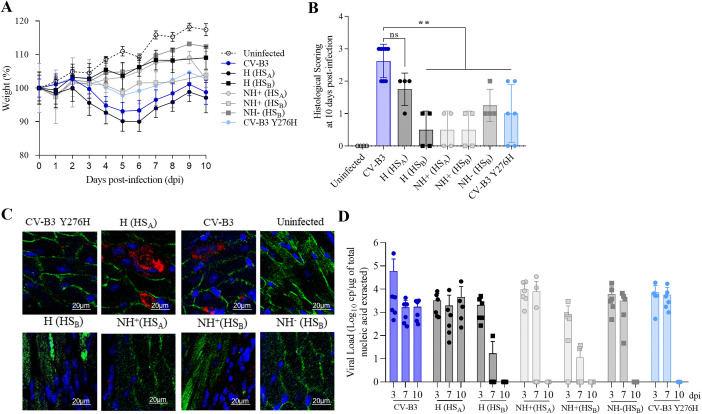
Characterization of cardiovirulence and genomic RNA replication of CV-B3 recombinants, 3Dpol mutants, and parental CV-B3 in DBA/2J mice hearts. **(A)** Weight loss in DBA/2J mice infected with 10^6^ PFU/mice of parental CV-B3 (*n* = 15), homologous (HS_A_, HS_B,_ *n* = 5–10) or non-homologous (NH^+^ (HS_A_, and HS_B_) or NH^−^, *n* = 5–10) recombinants, or the 3Dpol Y276H-mutant CV-B3 strain (*n* = 15), and in uninfected controls (*n* = 3), expressed as mean ± s.e.m. for the indicated number of mice. **(B)** Histological scores of cardiac lesions in mice infected with the indicated viruses (*n* = 4–6) or in uninfected controls (*n* = 3) at 10 days post-infection. Data shown as mean ± SD; **P < 0.01 by Mann–Whitney *U* test. **(C)** Immunofluorescence staining of viral capsid protein VP1 (red) and dystrophin (green) in heart sections (nuclei in blue) from mice infected with the indicated viruses or left uninfected at 10 days post-infection: magnification ×20. The images are representative of three mice per group. **(D)** Viral RNA loads in heart tissue, quantified by qRT-PCR, in mice infected with recombinant viruses, the CV-B3 Y276H mutant, or parental CV-B3 (*n* = 4–6) at the indicated time points post-infection (3, 7, and 10 days post-infection). CV-B3: Coxsackievirus B3, H: homologous, HS: Hot spot, NH-: non-homologous (deletion), NH + : non-homologous (insertion), PCR: polymerase chain reaction, RT: Reverse Transcription, VP1: viral protein 1.

To assess the cardiovirulence of recombinant and mutant group-B enteroviruses, DBA/2J mice were infected intraperitoneally with parental CV-B3, homologous (HS_A_/HS_B_) or non-homologous (NH) recombinants, or the 3Dpol Y276H-mutated CV-B3 strain. All strains displayed cardiac tropism, but their virulence and replication capacities diverged significantly. Parental CV-B3 and homologous recombinant in HS_A_ induced a significant weight loss (up to 20% at 6 days post-infection (6 dpi); *P* < 0.05) compared to homologous recombinant in HS_B,_ non-homologous recombinants (NH), or the 3Dpol Y276H mutant (Fig 5A).

Histopathological analysis at 10 dpi (acute phase) revealed myocarditis lesions in all groups, but median lesion scores were significantly higher in mice infected with parental CV-B3 or homologous recombinant (HS_A_) (*P* < 0.01 vs. H (HS_B_) and NH recombinants or 3Dpol Y276H mutant; Fig 5B). Viral replication activities in cardiac tissue correlated with histological severity at 10 dpi. VP1 capsid protein was detected exclusively in hearts of mice infected with parental CV-B3 or homologous recombinant (HS_A_), where it colocalized with regions of dystrophin disruption (Fig 5C). Consistent with this, viral RNA loads of one of the homologous recombinants (HS_B_) or non-homologous recombinants and 3Dpol Y276H mutant were undetectable in cardiac tissue at 10 dpi, suggesting rapid viral genomes clearance during acute infection. This contrasts with the persistent viral RNA loads of parental and homologous (HS_A_) strains (Fig 5D). The absence of detectable virus in NH and 3Dpol mutant-infected hearts coincided with reduced dystrophin disruption and milder histopathology, implying that defective recombination or 3Dpol activity may attenuate cardiac damage by limiting viral genomic RNA maintenance in cardiac tissues.

### 5′UTR recombination governs 5′TD RNA population dynamics and the host innate immunity interplay

**Fig 6:**
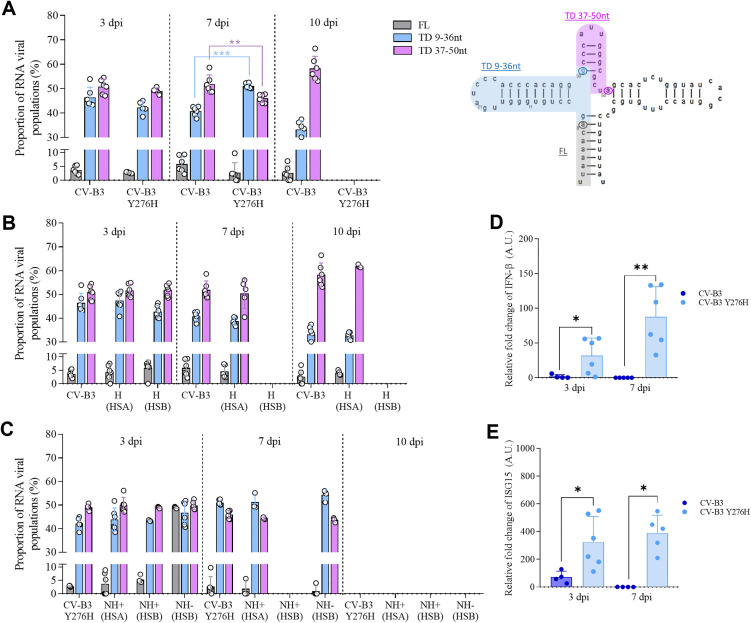
Emergence and maintenance of 5′TD CV-B3 populations in heart tissues of DBA/2J mice. **(A–C)** Proportions of full-length (FL) and 5′-terminally deleted (5′TD) CV-B3 RNA populations were measured by RACE-PCR and micro-electrophoresis (Agilent) in mice heart tissues at 3-, 7-, and 10-days post-infection (*n* = 3-6). Data represent mean ± SD. Statistical significance: **P < 0.01, ***P < 0.001 by two-way ANOVA with multiple comparisons. (A) [Left] Comparison of CV-B RNA population proportions in mice infected with 3Dpol Y276H mutant or parental CV-B3 strains. [Right] Schematic cloverleaf structure of CV-B showing positions of 8, 36, and 50 nt deletions: FL populations (gray, 0–8 nt deletions), 5′TD 9–36 nt populations (blue, 9–36 nt deletions), and 5′TD 37–50 nt populations (pink, 37–50 nt deletions). (B) Comparison of RNA population proportions in mice infected with homologous recombinants and parental CV-B3 strain. (C) Comparison of RNA population proportions in mice infected with non-homologous recombinants and 3Dpol Y276H mutant CV-B3 strain. **(D** and **E)** IFN-β and ISG15 mRNA expression reported as fold change relative to uninfected mice, normalized to housekeeping gene expression, in hearts infected with 3Dpol Y276H mutant and parental CV-B3 strains at 3- and 7-days post-infection. Data represent the mean ± SD. *: *P* < 0.05; **: *P* < 0.01 by Mann Whitney *U* test. 3Dpol: 3D polymerase, CV-B: Coxsackievirus, dpi: days post-infection, FL: full-length, H: homologous, HS: Hot spot, NH-: non-homologous (deletion), NH + : non-homologous (insertion), TD: 5′terminally deleted.

Since our team previously demonstrated that major pathogenic 5′ terminally deleted (5′TD) RNA populations modulate type I interferon (IFN-I) responses in murine myocarditis [1], we investigated how 3Dpol-mediated copy-choice recombination governs 5′TD RNA population dynamics and the host innate immunity interplay. Using established protocols [1], we quantified three distinct viral RNA populations in cardiac tissue during murine acute myocarditis: FL corresponding to FL genomes or genomes carrying deletions of up to 8 nucleotides; TD9–36nt corresponding to genomes with deletions ranging from 9 to 36 nucleotides; and TD37–50nt corresponding to genomes with deletions ranging from 37 to 50 nucleotides. (Figs 6A and S2). At 7 dpi, wild-type (WT) CV-B3 and homologous H (HS_A_) recombinants maintained stable major/minor 5′TD RNA ratios (Fig 6B). In contrast, the 3Dpol Y276H mutant significantly disrupted this equilibrium at 7 dpi (*P* < 0.05; Fig 6A). At the same time (7 dpi), viral RNA was longer detectable for the homologous H (HS_B_) recombinants, whereas residual RNA levels were still observed in the non-homologous NH+ (HS_A_ and HS_B_) recombinants (Fig 6B and 6C). To investigate the impact of 5′TD RNA population dynamics on innate immune responses in murine myocarditis, IFN-β, ISG15 mRNA levels were quantified in cardiac tissues. Significant variations were observed at 7 dpi, coinciding with the shift in the proportions of 5′TD CV-B3 RNA forms (TD50/TD15) in the myocardium of mice infected with recombination-deficient CV-B3 (3Dpol Y276H) (Fig 6D and 6E).

These results indicate that deficiency in 3Dpol recombination shifts the 5′TD viral population ratios toward viral RNA forms sensed by the immune system, leading to increased cardiac IFN-β and ISG15 expression and accelerated viral clearance in cardiac tissues, as shown in Fig 5. Collectively, these findings demonstrate that 3Dpol-dependent recombination activity maintains a balanced population of pathogenic 5′TD RNA forms (TD50), which modulate type I interferon responses to promote viral persistence in the heart. Disruption of this balance due to defective polymerase activity triggers activation of the type I IFN pathway in cardiac tissues, supporting the hypothesis of a mechanistic link between the equilibrium of 5′TD viral RNA forms and evasion of host innate immune defenses.

### 5′TD CV-B3 RNA proportions modulates type I interferon pathway activation in human cardiomyocytes

**Fig 7:**
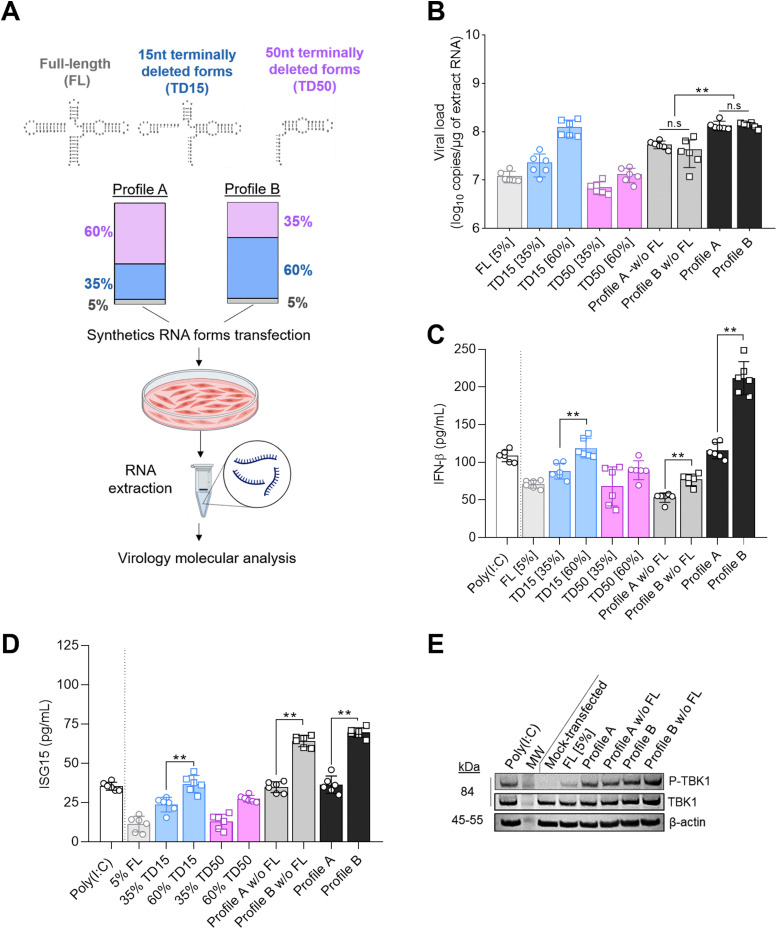
Impact of 5′TD CV-B3 populations on IFN-β pathway activation in human cardiomyocytes. **(A)** Experimental design: Full-length CV-B3, 5′TD with 15 nt deletions (TD15), and 5′TD with 50 nt deletions (TD50) synthetic RNAs were transfected into human cardiomyocytes (HCM), mimicking the proportions of FL and 5′TD RNA populations observed in mouse hearts infected with parental CV-B3 (Profile A) or 3D^pol^ Y276H-mutant CV-B3 (Profile B) at 7 days post-infection. **(B)** Viral RNA replication activity in HCM at 24 h post-transfection, assessed by RT-qPCR. **(C, D)** IFN-β and ISG15 protein levels in HCM supernatants at 24 h post-transfection, quantified by ELISA. **(E)** HCM cells were transfected with synthetic RNA and incubated up to 24 h for western blot analysis of TBK1/NAK phosphorylation and total TBK1/NAK. Data represent the mean ± SD for three independent experiments. *: *P* < 0.05; **: *P* < 0.01; ***: *P* < 0.001 by Mann–Whitney *U* test. The Poly(I:C) high molecular weight (HMW) was used as the positive control in these experiments.3Dpol: 3D polymerase, CV-B: Coxsackievirus, FL: full-length, MW: molecular weight, TD: 5′terminally deleted. RNA extraction: Created in BioRender. GLENET, M. (2026) https://BioRender.com/46p0qww. Petri dish: Created in BioRender. GLENET, M. (2026) https://BioRender.com/3dpmf05.

Since inhibition of 3Dpol-dependent recombination events disrupts 5′TD CV-B3 RNA form proportions, restoring IFN-β signaling pathway activation in our mouse myocarditis model (Fig 6), we postulated that the observed variations in major 5′TD and minor RNA forms proportions directly modulate the type I IFN activation pathway. To test this hypothesis, we transfected human cardiomyocytes (HCM) with two distinct RNA population mixes (Fig 7, Profiles A and B) as well as each of the three representative single viral RNA forms (Fig 7A). All synthetic CVB-5′TD RNA forms contain a 5′-terminal hammerhead ribozyme, generating authentic CV-B3 sequences without 5′ tri-phosphorylation, a known trigger of RIG-I-mediated IFN-I response [1].

At 24 h post-infection, quantification of viral RNA load in HCMs (Fig 7B) revealed no significant difference between the two distinct RNA population mixes (Profiles A and B). Notably, increasing the proportion of TD15 species (representative model of the 5′TD9–36 population), either alone or in combination, significantly elevated type I IFN and Interferon-stimulated-genes 15 kDa (ISG15) cytokine secretion in HCM supernatants (Fig 7C and 7D).

To further examine IFN pathway activation, lysates from HCMs transfected with full-length (FL) or 5′TD RNA forms (TD15, TD50, respectively, representative of 5′TD9-36nt and 5′TD37-50nt populations) were probed for TANK-binding kinase 1 (TBK1) phosphorylation by immunoblotting. As shown in Fig 7E, 5′TD RNA Profile A induced lower TBK1 phosphorylation than Profile B. Collectively, these data indicate that modulation of TD15 proportions in HCMs primarily governs type I IFN pathway activation during CV-B3 infection.

## Discussion

CV-B strains harboring genomic 5′ terminal deletions up to 50 nucleotides (5′TD) have been characterized alone or associated with minor cooperative full-length (FL) CV-B RNA forms in cardiac tissues from patients with acute or chronic cardiomyopathies [3,10,11,13]. Viral molecular mechanisms between 5′TD and FL RNA forms involved in the development of cardiomyocytes infection could be linked to genetic recombination mechanisms occurring in the 5′ UTR [10]. Our study demonstrates that 3Dpol-mediated copy-choice recombination in the 5′UTR dynamically regulates the FL/5′TD RNA form replication balance in cultured primary human cardiomyocytes (HCMs). Comparative infection studies in mice using CV-B3 WT and 3Dpol Y276H mutant demonstrate that 3Dpol actively maintains major 50-nt 5′TD RNA populations through copy-choice recombination events, thereby subverting innate immunity to sustain cardiac viral replication. Transfection experiments in HCMs revealed a length-dependent functional dichotomy: while 50-nt 5′TD populations evade innate immune detection, 9–36-nt 5′TD populations restore type I IFN pathway activation, consistent with in vivo observations. Our findings establish that group-B enterovirus 3D polymerase remodels viral populations through 5′UTR recombination to subvert cardiac type I interferon responses, explaining key mechanisms underlying the development of acute and chronic CV-B cardiac infections that drive human cardiomyopathies.

To define constraints on viral genetic exchanges during 5′-/3′- partner recombination in HCMs, we mapped recombination breakpoints in the 5′UTR, identifying two classes of recombinants (Fig 1): hotspots HS_A_ (spacer 1) and HS_B_ (spacer 2), consistent with prior reports [19,25,28]. HS_A_ allowed homologous recombination and limited non-homologous events (with nucleotide duplications) but required spacer 1 lengths ≥11 nucleotides (S2 Table with all recombinants sequences), suggesting structural plasticity constrained by essential roles in RNA replication/translation. In contrast, HS_B_ (spacer 2, ≈100 nucleotides) showed no such restrictions, likely due to its position between the IRES (SL-VII) and VP4 start codon [29–31]. This tolerance aligns with both spacers being non-coding regions flanked by structured/functional domains (VP4-VP2), where recombination viability depends on preserving adjacent essential elements [18,32,33]. Collectively, spacer 2 accommodated more sequence changes (particularly deletions), while spacer 1 length conservation appeared critical for viral viability.

Consistent with prior studies [25,21], viral recombinants showed significantly reduced fitness versus parental strains, with decreased genomic replication and virion production in HCMs (Fig 2). These observed variations could depend on 5′UTR recombination site location, potentially disrupting 5′UTR functional RNA secondary structures [1–3]. Such events may impair ribonucleoprotein complex (RNP) formation by altering the proper assembly of viral and host factors required for efficient replication complex formation at the 5′ end of the genome. This disruption may in turn affect the balance between positive- and negative-strand RNA synthesis, thereby reducing the efficiency of single-stranded positive-sense viral genomic RNA production. However, this last hypothesis has yet to be explored.

Combined proteomic and interactome approaches could identify factors binding 5′UTR during positive-strand synthesis [34] and could provide a better understanding of the observed fitness variation levels between parental and non-homologous recombinant viral strains.

To determine the recombination mechanism, we introduced 3Dpol mutations (including Y276H) into the 3′ partner. Y276H significantly reduced recombinant formation, confirming copy-choice as the dominant mechanism for 5′TD/FL genomic RNA modular exchanges (Fig 3). Nanoluciferase-tagged RNAs revealed asymmetric cooperativity: FL RNA enhanced 5′TD replication, while 5′TD forms suppressed FL replication via recombination (Fig 4). The combination of these two antagonist effects occurring at differential intensity levels may explain the helper effect of the FL on 5′TD forms and the absence of emergence of the FL viral populations during viral replication cycles in target cells. These findings may explain the detected imbalance of major deleted forms associated with minor FL viral RNA forms in target tissues during the natural course of EV-B infection in mice or humans [10,11,13].

In immunocompetent DBA/2J mice, all tested recombinant CV-B3 strains, including the 3Dpol Y276H mutant, exhibited a cardiac virulent phenotype (Fig 5A and 5B). Remarkably, viral RNA was completely cleared at day 10 post-infection in both the Y276H mutant and the homologous/non-homologous recombinants (Fig 5C and 5D). These results suggest that HS_B_ homologous and all non-homologous recombinants phenotypically copy the recombination-deficient 3Dpol mutant *in vivo* and share important pathophysiological features such as transient infectivity and rapid viral RNA clearance. Considering recent evidence that large 5′-terminally deleted (5′TD) RNA populations modulate the type I IFN activation pathway in mouse myocarditis [1], we investigated how 3Dpol-dependent recombination shapes 5′TD RNA populations to influence cardiac type I IFN signaling during CV-B3 cardiac infection (Fig 6). Comparative infection studies in mice with wild-type (WT) CV-B3 and the Y276H mutant have shown that 3Dpol dynamically regulates the ratio of 5′ terminal deletion (5′TD) RNA forms and promotes the emergence of a 49-nucleotides 5′TD variant (TD50). This TD50 RNA form promotes innate immune evasion and supports viral replication in cardiac tissue (Fig 6), supporting our recently published experimental results [1]. Further *in vivo* analysis linked shifts in shorter 5′TD RNA forms (9–36 nt deletions, TD15) to activation of the type I IFN pathway (Fig 7). Remarkably, these TD15 viral populations retain immunomodulatory stem-loop c and d structures in the genomic RNA domain-I of the 5′UTR, which may be sensed by RLRs or TLR3 in cardiomyocytes, as recently shown [1–3]. Consistent with this, transfection of human cardiomyocytes (HCM) with synthetic FL or various 5′TD CV-B3 RNA forms showed that TD15-enriched populations robustly activate IFN-β and ISG15 mRNA expression and secretion in our cultured cell model (Fig 7). ISG15 is known to suppress CV-B3 replication in human cardiomyocytes by directly inhibiting 2A protease activity, thereby preventing eIF4G cleavage and abrogating host translation. Consistent with these mechanisms, both the mRNA and protein of ISG15 were reported to be upregulated in heart biopsies from myocarditis patients and viral replication was reduced in functional studies in cell culture models [35,36].

Crucially, the Y276H mutation attenuates recombination, limiting the early emergence of immune evasive TD50 RNA forms, while favoring TD15 RNA forms that enhance antiviral innate immunity (Figs 6 and 7). This enhances viral clearance and reduces histopathologic damage to cardiac tissue, underscoring the role of recombination in the emergence of adaptive 5′TD major variants [18]. Our results demonstrate for the first time that 3Dpol-driven 5′UTR recombination dynamically remodels viral RNA populations to subvert cardiac type I IFN responses and highlight a key molecular mechanism that regulates the development of acute and persistent CVB3-induced cardiomyopathies. These findings emphasize the therapeutic potential of targeting 3Dpol to restrict recombination in group-B EV infections. By shifting the balance of the 5′TD population, reducing immune evasive TD50 RNAs while enhancing IFN-activating TD15 forms, existing or novel EV-B 3Dpol inhibitors (e.g., remdesivir or molnupiravir) could improve clinical outcomes in acute and chronic human myocarditis caused by group B EVs [11,13,37,38].

In summary, our results reveal a paradigm linking enterovirus recombination and subversion of cardiac innate immunity. We provide compelling evidence that 3Dpol-mediated copy-choice recombination remodels populations of 5′-terminally deleted (5′TD) viral RNAs, and thereby influences infection outcomes: the large 5′TD RNAs escape type I IFN sensors and support viral replication, whereas smaller populations of 5′TD RNAs with shorter deletions activate antiviral responses of the innate immune system and contribute to virus clearance. Thus, we show that recombination of the 5′UTR controls the pathogenesis of myocarditis through deletion length-dependent effects of 5′-RNA genomes on type I IFN responses, which are a central mechanism of CV-B heart infection. Therapies targeting 3Dpol-driven recombination and promoting immunostimulatory versus immune evasive viral RNA populations may represent promising strategies to combat enterovirus-induced acute and chronic cardiomyopathies.

## Materials and methods

### Ethic statement

Experiments were performed according to recommendations of the “National Commission of Animal Experiment (CNEA)” and the “National Committee on the Ethic Reflexion of Animal Experiments (CNREEA)”. The protocol was approved by the committee of animal experiments of the Reims Champagne-Ardenne (accreditation number #56), and then by the Ministry of Advanced Education and Research (permits #49400-2024031115337756). All efforts were made to minimize suffering in accordance with the Guide for the Care and Use of Laboratory Animals of the Direction des Services Vétérinaires, the French regulations to which our animal care and protocol adhered.

### Cultured cells

**Human primary cardiomyocytes** (HCM, ScienCell Research Laboratories, USA), originally derived from human heart tissue from both male and female fetal donors at 18–19 days of gestation, were used in this study. These cells were cultured in Cardiac Myocyte Medium-serum free (CMM-sf, ScienCell Research Laboratories, USA) supplemented with 10% FBS (Fetal Bovine Serum, Capricorn Scientific, Germany), 1% cardiac myocyte growth supplement (ScienCell, Clinisciences, USA), and 1% penicillin/streptomycin (ScienCell, Clinisciences, USA). HCM were cultured at 37 °C with 5% CO_2_.

**HeLa-229** cells were grown in Minimal Eagle’s essential Medium (MEM, Gibco, ThermoFisher, France) supplemented with 10% FBS (Capricorn Scientific, Germany), 2 mM L-glutamine (ThermoFisher, France), and 1% penicillin/streptomycin (ThermoFisher, France). HeLa-229 cells were cultured at 37 °C with 5% CO_2_.

### Plasmids construction

#### Coxsackievirus B3 strain 28 cDNA clones.

Coxsackieviruses B3/28 FL (CV-B3) (GenBank Accession AY752944.2) and deleted of the first 50 nucleotides at the 5′end (3′ partner) plasmid used in this study have been previously described [11,13].

#### Coxsackievirus B6 strain Schmitt cDNA cloning.

Two cDNA, encoding Coxsackievirus B6/Schmitt (ATTC VR-155) FL (CV-B6) and deleted of 3′ region after VP4 coding sequence RNA genomes (5′ partner) have been generated. CV-B6 RNA genome (GenBank Accession AF105342.1) was isolated from viral supernatant by QIAamp Viral RNA Mini Kit (Qiagen, France). Viral RNA was then reverse transcribed using SuperScript II kit (Invitrogen, ThermoFisher, France) with 3′EndPolyT primer (S1 Table). cDNA was processed by two steps directed mutagenesis allowing addition of a hammer-head ribozyme feature upstream of the first nucleotides of the viral sequence and MluI restriction site downstream viral genome (S1 Table). PCR products were cloned into the vectors pCR-XL-TOPO and pCR2.1 by PCR cloning kits (Invitrogen, ThermoFisher, France) for CV-B6 and 5′ partner respectively.

### Experimental recombination model for assessment of genomic RNA exchanges within the 5′-UTR region

We developed an experimental recombination model based on a previously established assay [25] to assess genomic RNA exchanges within the 5′-UTR region of group-B enteroviruses in primary human cardiomyocytes (HCM). CV-B3/28 and CV-B6/Schmitt (CV-B6) were selected due to their 86% nucleotide identity in the 5′-UTR (S1A Fig), enabling identification of RNA recombination events through Sanger sequencing. The 3′ partner was constructed as CV-B3/28 RNA with a deletion of the first 50 nucleotides in the 5′ UTR (S1B Fig); while the 5′ partner was constructed as CV-B6/Schmitt RNA containing the complete 5′ UTR followed only by the VP4 sequence (S1B Fig). The CVB6 partner is non-infectious, whereas the TD50 genome exhibits only minimal infectivity and cannot independently produce detectable plaque-forming virus. Upon co-transfection into human cardiomyocytes (HCM) at a ratio of 19:1, mimicking the proportions of EV-B RNA forms detected in human cardiac tissues [10], genome rescue was evidenced by the presence of infectious recombinants (S1C Fig).

#### Homologous and non-homologous recombinant cloning.

The homologous (H) recombinant cDNA was constructed with nucleotides 1–64 of CV-B6 followed by CV-B3 sequence from position 65 onward, while non-homologous recombinants included: a deletion mutant (NH-) featuring nucleotides 1–649 of CV-B6 joined to nucleotides 711–end of CV-B3, and a duplication mutant (NH+) containing nucleotides 1–198 of CV-B6 fused to nucleotides 51–end of CV-B3 (S1 Table). For H and NH- recombinants, CV-B6 sequences were PCR-amplified and cloned into pCR-XL-TOPO vector, while CV-B3 segments were separately amplified (S1 Table), with both fragments assembled using the In-Fusion HD kit (Takara Bio). The NH+ recombinant was synthesized de novo and cloned into pCR-XL-TOPO by Genewiz (Azenta Life Sciences).

The nucleotide present at position 234 in the 5′UTR was verified for all parental strains and generated recombinant viruses by complete genome sequencing. The presence of a uridine (U) at nucleotide position 234, a marker associated with virulent CVB3 strains, was confirmed in all analyzed viruses.

#### Substitutions D79H, Y276H, and L421A in CV-B3/28 3D polymerase gene.

Each substitution was introduced into either the wild-type (WT) CV-B3/28 or the 3′ partner cDNA genomes using site-directed mutagenesis with primers listed in S1 Table.

#### Nanoluciferase-tagged CV-B3/28.

Nanoluciferase was introduced into the CV-B3 sequence upstream the VP4 coding sequence in the same open reading frame as the viral polyprotein and followed by viral 3C protease cleavage site [39]. Nanoluciferase-tagged CV-B3 sequence was synthesized and cloned into pCR-XL-TOPO vector by Genewiz (Azenta life Sciences, Germany). The deletion of the first 49 nucleotides of CV-B3 was performed as previously described [11,13] and substitution Y276H was introduced using primers in S1 Table.

#### *In vitro* transcription.

Plasmids encoding Coxsackieviruse B sequences have been linearized with restriction enzyme MluI. *In vitro* T7 transcription was performed by T7 RNA polymerase using MEGAscript T7 transcription kit (Invitrogen, ThermoFisher, France). Synthetic viral RNA was purified using MEGAclear Transcription Clean-Up Kit (Invitrogen, ThermoFisher, France) according to the manufacturer’s instructions.

#### Transfection of synthetic viral RNAs in HCM.

Cell monolayers were seeded in 6-well plates at a concentration of 8 × 10^5^ cells per well incubate overnight at 37 °C with 5% CO_2_. After wash step, cells were transfected with transfection mixtures comprised of 2.5 µg synthetic viral RNA, 1 mL of serum free medium in the presence of Lipofectamine 2000 according to the manufacturer’s instructions (Invitrogen, ThermoFisher, France). The mixtures were incubated 20 min at room temperature and were then added to the cells. Cells were incubated with transfection mixtures for 1 h at 37 °C with 5% CO_2._ After wash step, cells were incubated with 2 mL of serum free medium during indicated times.

#### Co-transfection recombination assays in HCM.

HCM cells were co-transfected with 5% of 5′ partner and 95% of 3′ partner (ratio 1:19) synthetic RNA (Fig 2) and incubated at 37 °C for 24 h with serum free DMEM. Following incubation, culture supernatants and infected cells were harvested together to recover both extracellular and intracellular viral RNA. Samples were subjected to three freeze-thaw cycles to release intracellular nucleic acids. The lysates were then clarified by centrifugation at 7,000 rpm for 10 min at 4 °C, and total viral RNA was subsequently extracted from the resulting supernatants.

#### Viral production by cellular transfection of synthetic viral RNAs.

HeLa cells were incubated at 37 °C for 72 h with 2 mL of serum free MEM. The supernatants were collected, cleared of cell debris by centrifugation at 7,000 rpm for 10 min at 4 °C and the concentration of viral production was measured by plaque-forming-unit assay (PFU) [10].

#### Isolation of CV-B recombinant viruses.

HeLa cells seeded in 6-well plates were infected with recombinant viruses generated from HCM transfections. After 1 h of incubation, wells were subsequently maintained under 4 mL of overlay composed 1% semi-solid agarose (ThermoFisher) in a 1X MEM, 2 mM L-Glutamine, 1% penicillin/streptomycin, and 0.25% sodium bicarbonate. The plates were incubated for 72 h at 37 °C with 5% CO_2_. Recombinants forming plaques were picked through agarose overlay and transferred in 160 µL of serum free supplemented DMEM. Recombinant viruses’ stocks were obtained after clarification by centrifugation at 7,000 rpm for 10 min at 4 °C [40].

#### Growth kinetics of recombinant viruses in HCM.

HCM cells were seeded at a density of 2 × 10^5^ cells per well in 24-well plates. Replication kinetics of parental and recombinant viruses were assessed by infection of HCM at MOI 10 (multiplicity of infection of 10). After wash step, cells were incubated with 500 µL of serum free DMEM at 37 °C for indicated times in the experiments.

#### Total viral and cellular RNA extraction.

Supernatant and cells were harvested following three freeze-thaw cycles. The lysates were cleared of cell debris by centrifugation at 7,000 rpm for 10 min at 4 °C. Total RNA was extracted with TRI-REAGENT (Euromedex, France) and was stored at −80 °C.

#### Detection and quantification of viral RNA by RT-qPCR.

Viral RNA copy number was quantified using one-step RT-qPCR with the qScript XLT 1-Step RT-qPCR ToughMix (QuantaBio) on a CFX96 Touch system (Bio-Rad). Reactions employed CVB3/28-specific primers and a FAM/TAMRA-labeled probe (Eurogentec, Belgium) [11]. Thermal cycling: reverse transcription at 50 °C for 10 min; denaturation at 95 °C for 1 min; 45 cycles of 95 °C for 10 s and 63 °C for 30 s. Viral load was calculated as genome copies per microgram (cp/μg) of extracted nucleic acid.

#### 5′UTR nucleotide sequencing of viable recombinant viruses.

cDNA was synthesized using the Superscript II Reverse Transcriptase kit (Invitrogen, Thermo Fisher, France) with random hexamer primers in a 20 µL reaction volume, following the manufacturer’s protocol. A two-step semi-nested PCR was then performed using the KAPA Taq Polymerase kit (Clinisciences, France) with primers listed in S1 Table. Amplified DNA bands were gel-purified (QIAquick Gel Extraction Kit, Qiagen) and Sanger-sequenced by Genewiz (Azenta Life Sciences, Germany). Sequences were analyzed and aligned to parental references using CLC Main Workbench v7.6.3 (QIAGEN, France). All recombinant 5′UTR sequences are provided in S2 Table.

### Positive and negative strand RNA ratios

#### Positive and negative-strand RNA ratio quantification.

Negative-strand RNA was isolated from total RNA by annealing a biotinylated strand-specific primer (E3REV; 5′-GGAACCGACTACTTTGGGTGTCCGTG-3′) followed by capture with streptavidin magnetic beads (Invitrogen) (S1 Table). Both purified negative-strand RNA and total viral RNA were quantified using One-Step real-time RT-PCR with standard curves generated from serial transcript dilutions, and the positive/negative strand ratio was calculated as: (total EV RNA - negative strand EV RNA)/ negative strand EV RNA [41].

#### Quantitative characterization of CVB-TD by RACE-PCR and NGS.

RACE-PCR assays were performed as previously described [11]. Amplification products were simultaneously sized and quantified using the Agilent High Sensitivity DNA Kit on a Bioanalyzer (Agilent, France), following the manufacturer’s instructions. For *in vivo* samples, CVB-TD populations were quantitatively characterized by RACE-PCR and subsequently validated by NGS as referenced [1]. Viral populations with deletions of fewer than 8 nucleotides were classified as FL [10,13].

### Nanoluciferase reporter assay

HCM cells were seeded in 24-well plates at a concentration of 2 × 10^5^ cells per well and incubated overnight at 37 °C with 5% CO_2_. Cells were transfected with 100 ng synthetic viral RNA complexed with Lipofectamine 2000 (Invitrogen, Thermo Fisher Scientific) in 100 µL serum-free medium, per the manufacturer’s protocol. Following a 20-min incubation at RT, transfection mixtures were added to cells and incubated for 1 h (37 °C, 5% CO_2_). After washing, cells were maintained in 500 µL serum-free medium.

At 24 h post-transfection, supernatants were discarded, and cells were lysed with 1X Passive Lysis Buffer (Promega, France). Lysates were mixed 1:1 (v/v) with Nano-Glo Luciferase Reagent (prepared by combining Nano-Glo Substrate and Buffer; Promega). Luminescence was quantified using a Varioskan LUX multimode reader (Thermo Fisher Scientific) and normalized to total protein concentration.

#### Western blot.

Protein extraction was performed 8 h post-transfection of HCM cells (2 µg of synthetics RNA in 6-well plates, 37 °C, 5% CO_2_), with lysis in 120 µL RIPA buffer (Thermo Scientific) including vortexing every 15 min for 1 h at 4 °C. Lysates were centrifuged (7,400 *g*, 15 min, 4 °C), and supernatants were mixed with 1X NuPAGE LDS Sample Buffer and 1X NuPAGE Reducing Agent (Invitrogen), then denatured (70 °C, 10 min). Proteins were resolved on NuPAGE 4–12% Bis-Tris gels (Invitrogen) and transferred to nitrocellulose membranes (GE Healthcare) using a Trans-Blot SD Semi-Dry Transfer Cell. After blocking with 5% BSA, membranes were probed with anti-Phospho-TBK1/NAK (Ser172) (D52C2) (1:1000, #5483, Cell Signaling Technology), anti-TBK1/NAK (D1B4) (1:1000, #3504, Cell Signaling Technology), anti-eIF4G (1:1000, #2469S, Cell Signaling Technology), anti-VP1 (1:1000, #M47, Cox mAB 31A2, Mediagnost), and anti-β-actin (1:1000, #3700, Cell Signaling Technology), followed by HRP-conjugated secondary antibodies: anti-rabbit (1:10,000, #7074S, Cell Signaling Technology), and anti-mouse (1:5,000, NA9310V, GE Healthcare). Signal detection used Pierce ECL, SuperSignal West Pico PLUS, and SuperSignal West Atto Ultimate substrates (Thermo Scientific) on an iBright CL1500 Imaging System.

#### Infection of DBA/2J mice.

Five-week-old male DBA/2J mice (purchased from Charles River Laboratory, France) were housed under pathogen-free conditions. Mice were infected intraperitoneally with 10^6^ Pfu/mice in 150 µl of physiological serum; uninfected mice were inoculated with 150 μl of physiological serum. Loss of body weight was monitored daily for 10 days post-infection and mice were euthanized if they had ≥20% loss of their initial body weight, according to our protocol. Mice were euthanized by cervical dislocation before heart collection at various time points post-infection (3-, 7-, and 10-days post-infection). Heart were taken and fixed in formaldehyde 4% for histology or homogenized in 600 µl of PBS solution and placed in the tissue lyser (Tissue Lyser LT, Qiagen). After centrifugation at 12,000 *g* for 15 min at 4 °C, the supernatant was recovered, and the remaining unground tissue was incubated for 1 h at 56 °C in TrisHCl buffer (20 mM) and 0.5% SDS in the presence of 20 µl of proteinase K with regular stirring. The tissue lysate was then used for nucleic acid extraction.

### Histological analysis and immunofluorescence staining

#### *In vitro* samples.

HCM cells were plated at a density of 2 × 10^5^ cells per well in 24-well plates and were infected at MOI 10 as described above. At 24 h post-infection, cells were fixed with 4% paraformaldehyde. Immunofluorescence staining was performed as previously described [13]. 1:300 diluted rabbit anti-dystrophin (Abcam, UK, ab15277), and 1:500 diluted mouse anti-viral protein 1 (VP1; Dako, 5D8/1) antibodies were used. Secondary antibodies were conjugated with AlexaFluor514 for anti-rabbit and AlexaFluor633 for anti-mouse (Thermofisher, France). DAPI (Sigma, USA) was used to stain cell nuclei. Confocal images were acquired using a Zeiss confocal microscope (PICT Platform, University of Reims).

#### Heart samples.

Four-μm-thick paraffin sections were cut at three levels. The slides were stained with Hematoxylin, Eosin, and Safran (HES, Pathology department, Reims, France) for histological scoring. The histological lesions were graded using a scale of 0 (no necrosis or inflammation), 1 (1 or 2 foci by slice), 2 (1 or 2 foci on every slice), and 3 (more than 2 foci on every slice) [42]. Immunofluorescence staining for viral capsid protein VP1 and cell dystrophin was performed as previously described [1,13].

### Phylogenic analysis

Enterovirus strains were aligned using Clustal W version. Evolutionary distances were calculated using the kimura 2-parameter method. The tree was constructed using the mixed-linear method as implemented in MEGA 7 software. Bootstrap values from 1,000 replicates are shown at the nodes. Scale bar indicates the genetics distance.

### Statistical analysis

All data are expressed as mean ± SD. Statistical comparison was performed on at least three independent experiments. Quantitative variables were compared using Mann–Whitney *U* and Fischer’s exact test. The value of *P* < 0.05 was considered significant. Statistical analyses were conducted using GraphPad Prism version 11.0.2 (GraphPad) software.

## Supporting information

S1 Fig Group-B enterovirus genome organization. **(A)** Enterovirus-B viral genome is composed of two ORFs which one is a polyprotein coding for capsidic (Viral proteins 1–4) and non-structural viral proteins such as proteases 2A, 3C and the 3D polymerase. This long ORF is flanked by two untranslated regions: the 5′UTR is composed of a Cloverleaf-like secondary structure (CL, Domain I) responsible for the viral genomic replication separated by a spacer sequence comprising two C-rich clusters from an Internal Ribosome Entry Site element type 1 (IRES, stem-loops II to VII) which has a role in the viral translation. **(B)** Schematic representations of the predicted 5′end Cloverleaf secondary structures (CL, Domain I) of the enteroviral positive strand RNA. The full-length (FL) cloverleaf is composed of a stem “a” (nucleotides 2–9 with 80–87), stem-loop “b” (nucleotides 10–34), stem-loop “c” (nucleotides 35–45) and a stem-loop “d” (nucleotides 46–79). The TD 9–36nt cloverleaf lost the 9 first nucleotides and the TD37–50nt cloverleaf lost the 36 first nucleotides. TD: terminally deleted forms.
(TIF)

S2 Fig 5′UTR recombination model of EV-B in human cardiomyocytes (HCM). **(A)** Molecular phylogenetic analysis of EV-B based on alignment of 5′UTR sequences using the maximum likelihood method. Robustness was evaluated by 1,000 bootstrap iterations; percentages indicate the occurrence of corresponding clusters. The selected EV-B partners (CV-B3/28, green; CV-B6/Schmitt, blue) share 86% nucleotide identity in the 5′UTR. **(B)** The CV-B6/Schmitt 5′ partner (green) comprises the complete 5′UTR followed by the VP4 sequence. The 3′ partner (TD50 CVB3/28, blue) consists of the CV-B3/28 sequence lacking the first 49 nucleotides. **(C)** Infectious virus titers in HCM following transfection of parental full-length RNA, 5′ partner (5′P), 3′ partner (3′P), alone or in combination (1:19 ratio, 5′P × 3′P) at 24 h post-transfection. **(D)** Titers of infectious recombinants after isolation by plaque picking and amplification in HCM at 72 h post-infection. Data represent the mean ± SD. **: *P* < 0.01; ***: *P* < 0.001; ****: *P* < 0.0001 by Mann–Whitney *U* test. 5′P: 5′ partner, 3′P: 3′ Partner, EV: Enterovirus, CV-B: Coxsackievirus B, UTR: Untranslated region, TD50: Terminal deletion of 50 nucleotides, Rec: recombinant.
(TIF)

S3 Fig Quantitative detection of full-length (FL) and 5′ terminally deleted (5′TD) CV-B populations in heart tissues of infected DBA/2J mice. **(A–C)** Proportions of FL and 5′TD CV-B3 RNA populations were measured using RACE-PCR followed by micro-electrophoresis (Agilent) in heart tissues collected at 3-, 7-, and 10-days post-infection. Data were analyzed using the Agilent 2100 Bioanalyzer with high sensitivity DNA kits. (A) Representative electropherograms of RACE-PCR products from synthetic positive controls: FL and 5′TD CV-B3 cDNAs; FL ranging between 99–107 bp, 5′TD 9–36 nt deletions between 72–98 bp, and 5′TD 37–50 nt deletions between 57–71 bp (±10% confidence interval). (B) Representative electropherograms from heart tissues of mice infected with parental CV-B3 and homologous recombinants. (C) Representative electropherograms from heart tissues of mice infected with 3Dpol-mutated CV-B3 strain and non-homologous recombinants. 3Dpol: 3D polymerase, bp: base pair, CV-B: Coxsackievirus, dpi: days post-infection, FL: full-length, H: homologous, HS: Hot spot, NH-: non-homologous (deletion), NH + : non-homologous (insertion), TD: 5′terminally deleted.
(TIF)

S1 Table Primer sequences. Footprint: CV-B3, Coxsackievirus B3/28; CV-B6, Coxsackievirus B6/Schmitt; F, forward; HH, Hammerhead; HS, Hot spot; R, Reverse; Rec, Recombinant; H, Homologous; NH, non-homologous.
(XLSX)

S2 Table Recombinant sequences. Footprint: CV-B3, Coxsackievirus B3/28; CV-B6, Coxsackievirus B6/Schmitt; HS, Hot spot; Rec, Recombinant; H, Homologous; NH, non-homologous; NHS, non-hotspot.
(DOCX)

S1 Raw Image iBright CL1500 image analysis report and annotated Western blot indicating sample loading order. Human primary cardiomyocytes (HCMs) were transfected with synthetic RNA mimicking the proportions of full-length (FL) and 5′ terminally deleted (5′TD) RNA populations observed in mouse hearts infected with either parental CV-B3 (Profile A) or the 3Dpol Y276H mutant CV-B3 (Profile B) at 7 days post-infection. Following transfection, cells were incubated for up to 24 h before Western blot analysis of TBK1/NAK phosphorylation and total TBK1/NAK expression. The loading order was as follows: lane 1, high molecular weight (HMW) poly(I:C) used as a positive control; lane 2, molecular weight marker (PageRuler Prestained Protein Ladder, Thermo Fisher Scientific); lane 3, mock-transfected; lane 4, FL (5%); lane 5, Profile A; lane 6, Profile A without FL; lane 7, Profile B; lane 8, Profile B without FL.
(PDF)
