# Supplementary material for: EV-B 3D polymerase remodels viral populations through 5′UTR recombination to subvert cardiac antiviral innate immunity
Source: PLoS Pathog. 2026 Jul 24;22(7):e1014441. doi: 10.1371/journal.ppat.1014441 (PMC13426973; doi:10.1371/journal.ppat.1014441)
Supplement: S2 Table — Footprint: CV-B3, Coxsackievirus B3/28; CV-B6, Coxsackievirus B6/Schmitt; HS, Hot spot; Rec, Recombinant; H, Homologous; NH, non-homologous; NHS, non-hotspot. (DOCX) [file ppat.1014441.s005.docx]

| Homologous recombinants (Rec H) | | | | | |
| --- | --- | --- | --- | --- | --- |
|  | CV-B6/Schmitt | CV-B3/28 | Hot Spot | Sequence |  |
| 1.4 | 661 | 662 | HS_B_ | ttaaaacagc ctgtgggttg tacccaccca cagggcccac tgggcgctag cactctggta  ttgcggtacc tttgtgcgcc tgttttaccc accctccccc aacgtaactt agaagcctga  catatacggt cacaagccag cccagtacgc caactgggta ccgaccaagc acttctgtta  ccccggactg agtatcaata agctgccaaa gcggctgaag gagaaaacgt tcgttacccg  gccaattact tcgagaaacc cagtaacacc atgaaagttg cgtagcgttt cattcagcac  aatcccagtg tagatcaggt cgatgagtca ccgcattccc cacaggcgac tgtggcggtg  gctgcgttgg cggcctgccc atggggcaaa cccatgggac gcttcaatac tgacatggtg  cgaagagtct attgagctaa ttggtagtcc tccggcccct gaatgcggct aatcctaact  gcggagcagg cactcgcaga ccagcgagca gcttgtcgta atgggcaact ccgcagcgga  accgactact ttgggtgtcc gtgtttccta tttcctttac actggctgct tatggtgaca  attgagaaat tgttaccata tagctattgg agtggccatc cggtgactaa cagagcaata  aatatctctt tgttgggttt ataccactta gcttgaaaga ggttaaaaca ttacaattca  ttgttaagtt gaatacagca aaatgggagc tcaagtatca acgcaaaaga ctggggcaca  tgagaccggg ctgaatgcta gcggcaattc catcattcac tacacaaatg ttaattatta  caaggatgcc gcatccaact cagccaatcg gcaggatttc actcaagacc cgggcaagtt  cacagaacca gtaaaagata tcatgattaa atcactacca gctctcaact cccccacagt  agaggagtgc ggatacagtg acagggcgag atcaatcaca |  |
| 1.6 | 710 | 711 | HS_B_ | ttaaaacagc ctgtgggttg tacccaccca cagggcccac tgggcgctag cactctggta  ttgcggtacc tttgtgcgcc tgttttaccc accctccccc aacgtaactt agaagcctga  catatacggt cacaagccag cccagtacgc caactgggta ccgaccaagc acttctgtta  ccccggactg agtatcaata agctgccaaa gcggctgaag gagaaaacgt tcgttacccg  gccaattact tcgagaaacc cagtaacacc atgaaagttg cgtagcgttt cattcagcac  aatcccagtg tagatcaggt cgatgagtca ccgcattccc cacaggcgac tgtggcggtg  gctgcgttgg cggcctgccc atggggcaaa cccatgggac gcttcaatac tgacatggtg  cgaagagtct attgagctaa ttggtagtcc tccggcccct gaatgcggct aatcctaact  gcggagcagg cactcgcaga ccagcgagca gcttgtcgta atgggcaact ccgcagcgga  accgactact ttgggtgtcc gtgtttccta tttcctttac actggctgct tatggtgaca  attgagaaat tgttaccata tagctattgg agtggccatc cggtgactaa cagagcaata  atatatttgt ttgttggctt tgtgccactt aatttgaagg ttgttaagac ttacaattca  ttgttaagtt gaatacagca aaatgggagc tcaagtatca acgcaaaaga ctggggcaca  tgagaccggg ctgaatgcta gcggcaattc catcattcac tacacaaatg ttaattatta  caaggatgcc gcatccaact cagccaatcg gcaggatttc actcaagacc cgggcaagtt  cacagaacca gtaaaagata tcatgattaa atcactacca gctctcaact cccccacagt  agaggagtgc ggatacagtg acagggcgag atcaatcaca |  |
| 1.63 | 302 | 303 | NHS | ttaaaacagc ctgtgggttg tacccaccca cagggcccac tgggcgctag cactctggta  ttgcggtacc tttgtgcgcc tgttttaccc accctccccc aacgtaactt agaagcctga  catatacggt cacaagccag cccagtacgc caactgggta ccgaccaagc acttctgtta  ccccggactg agtatcaata agctgccaaa gcggctgaag gagaaaacgt tcgttacccg  gccaattact tcgagaaacc cagtaacacc atgaaagttg cgtagcgttt cattcagcac  aaccccagtg tagatcaggt cgatgagtca ccgcattccc cacgggcgac cgtggcggtg  gctgcgttgg cggcctgccc atggggaaac ccatgggacg ctctaataca gacatggtgc  gaagagtcta ttgagctagt tggtagtcct ccggcccctg aatgcggcta atcctaactg  cggagcacac accctcaagc cagagggcag tgtgtcgtaa cgggcaactc tgcagcggaa  ccgactactt tgggtgtccg tgtttcattt tattcctata ctggctgctt atggtgacaa  ttgagagatt gttaccatat agctattgga ttggccatcc ggtgaccaat agagctatta  tatatctctt tgttgggttt ataccactta gcttgaaaga ggttaaaaca ttacaattca  ttgttaagtt gaatacagca aaatgggagc tcaagtatca acgcaaaaga ctggggcaca  tgagaccggg ctgaatgcta gcggcaattc catcattcac tacacaaatg ttaattatta  caaggatgcc gcatccaact cagccaatcg gcaggatttc actcaagacc cgggcaagtt  cacagaacca gtaaaagata tcatgattaa atcactacca gctctcaact cccccacagt  agaggagtgc ggatacagtg acagggcgag atcaatcaca |  |
| 2.1 | 61 | 62 | HS_A_ | ttaaaacagc ctgtgggttg tacccaccca cagggcccac tgggcgctag cactctggta  tcacggtacc tttgtgcgcc tgttttatac cccctccccc aactgtaact tagaagtaac  acacaccgat caacagtcag cgtggcacac cagccacgtt ttgatcaagc acttctgtta  ccccggactg agtatcaata gactgctcac gcggttgaag gagaaagcgt tcgttatccg  gccaactact tcgaaaaacc tagtaacacc gtggaagttg cagagtgttt cgctcagcac  taccccagtg tagatcaggt cgatgagtca ccgcattccc cacgggcgac cgtggcggtg  gctgcgttgg cggcctgccc atggggaaac ccatgggacg ctctaataca gacatggtgc  gaagagtcta ttgagctagt tggtagtcct ccggcccctg aatgcggcta atcctaactg  cggagcacac accctcaagc cagagggcag tgtgtcgtaa cgggcaactc tgcagcggaa  ccgactactt tgggtgtccg tgtttcattt tattcctata ctggctgctt atggtgacaa  ttgagagatt gttaccatat agctattgga ttggccatcc ggtgaccaat agagctatta  tatatctctt tgttgggttt ataccactta gcttgaaaga ggttaaaaca ttacaattca  ttgttaagtt gaatacagca aaatgggagc tcaagtatca acgcaaaaga ctggggcaca  tgagaccggg ctgaatgcta gcggcaattc catcattcac tacacaaatg ttaattatta  caaggatgcc gcatccaact cagccaatcg gcaggatttc actcaagacc cgggcaagtt  cacagaacca gtaaaagata tcatgattaa atcactacca gctctcaact cccccacagt  agaggagtgc ggatacagtg acagggcgag atcaatcaca |  |
| 2.15 | 62 | 63 | HS_A_ | ttaaaacagc ctgtgggttg tacccaccca cagggcccac tgggcgctag cactctggta  ttacggtacc tttgtgcgcc tgttttatac cccctccccc aactgtaact tagaagtaac  acacaccgat caacagtcag cgtggcacac cagccacgtt ttgatcaagc acttctgtta  ccccggactg agtatcaata gactgctcac gcggttgaag gagaaagcgt tcgttatccg  gccaactact tcgaaaaacc tagtaacacc gtggaagttg cagagtgttt cgctcagcac  taccccagtg tagatcaggt cgatgagtca ccgcattccc cacgggcgac cgtggcggtg  gctgcgttgg cggcctgccc atggggaaac ccatgggacg ctctaataca gacatggtgc  gaagagtcta ttgagctagt tggtagtcct ccggcccctg aatgcggcta atcctaactg  cggagcacac accctcaagc cagagggcag tgtgtcgtaa cgggcaactc tgcagcggaa  ccgactactt tgggtgtccg tgtttcattt tattcctata ctggctgctt atggtgacaa  ttgagagatt gttaccatat agctattgga ttggccatcc ggtgaccaat agagctatta  tatatctctt tgttgggttt ataccactta gcttgaaaga ggttaaaaca ttacaattca  ttgttaagtt gaatacagca aaatgggagc tcaagtatca acgcaaaaga ctggggcaca  tgagaccggg ctgaatgcta gcggcaattc catcattcac tacacaaatg ttaattatta  caaggatgcc gcatccaact cagccaatcg gcaggatttc actcaagacc cgggcaagtt  cacagaacca gtaaaagata tcatgattaa atcactacca gctctcaact cccccacagt  agaggagtgc ggatacagtg acagggcgag atcaatcaca |  |
| 2.19 | 931 | 932 | NHS | ttaaaacagc ctgtgggttg tacccaccca cagggcccac tgggcgctag cactctggta  ttgcggtacc tttgtgcgcc tgttttaccc accctccccc aacgtaactt agaagcctga  catatacggt cacaagccag cccagtacgc caactgggta ccgaccaagc acttctgtta  ccccggactg agtatcaata agctgccaaa gcggctgaag gagaaaacgt tcgttacccg  gccaattact tcgagaaacc cagtaacacc atgaaagttg cgtagcgttt cattcagcac  aatcccagtg tagatcaggt cgatgagtca ccgcattccc cacaggcgac tgtggcggtg  gctgcgttgg cggcctgccc atggggcaaa cccatgggac gcttcaatac tgacatggtg  cgaagagtct attgagctaa ttggtagtcc tccggcccct gaatgcggct aatcctaact  gcggagcagg cactcgcaga ccagcgagca gcttgtcgta atgggcaact ccgcagcgga  accgactact ttgggtgtcc gtgtttccta tttcctttac actggctgct tatggtgaca  attgagaaat tgttaccata tagctattgg agtggccatc cggtgactaa cagagcaata  atatatttgt ttgttggctt tgtgccactt aatttgaagg ttgttaagac gctcaatctg  atactacttc taaataaagc aaaatgggag ctcaagtctc aacacagaaa acaggagctc  acgaaaccgc attaaacgcg cagggaaact cggtacttca ctacaccaac ataaattact  acaaggatgc ggcttccaac tcagcaaata ggcaggactt tacacaagac cccagcaaat  tcaccgagcc agttaaggat gtgatgataa atcactacca gctctcaact cccccacagt  agaggagtgc ggatacagtg acagggcgag atcaatcaca |  |
| 2.20 | 931 | 932 | NHS | ttaaaacagc ctgtgggttg tacccaccca cagggcccac tgggcgctag cactctggta  ttgcggtacc tttgtgcgcc tgttttaccc accctccccc aacgtaactt agaagcctga  catatacggt cacaagccag cccagtacgc caactgggta ccgaccaagc acttctgtta  ccccggactg agtatcaata agctgccaaa gcggctgaag gagaaaacgt tcgttacccg  gccaattact tcgagaaacc cagtaacacc atgaaagttg cgtagcgttt cattcagcac  aatcccagtg tagatcaggt cgatgagtca ccgcattccc cacaggcgac tgtggcggtg  gctgcgttgg cggcctgccc atggggcaaa cccatgggac gcttcaatac tgacatggtg  cgaagagtct attgagctaa ttggtagtcc tccggcccct gaatgcggct aatcctaact  gcggagcagg cactcgcaga ccagcgagca gcttgtcgta atgggcaact ccgcagcgga  accgactact ttgggtgtcc gtgtttccta tttcctttac actggctgct tatggtgaca  attgagaaat tgttaccata tagctattgg agtggccatc cggtgactaa cagagcaata  atatatttgt ttgttggctt tgtgccactt aatttgaagg ttgttaagac gctcaatctg  atactacttc taaataaagc aaaatgggag ctcaagtctc aacacagaaa acaggagctc  acgaaaccgc attaaacgcg cagggaaact cggtacttca ctacaccaac ataaattact  acaaggatgc ggcttccaac tcagcaaata ggcaggactt tacacaagac cccagcaaat  tcaccgagcc agttaaggat gtgatgataa atcactacca gctctcaact cccccacagt  agaggagtgc ggatacagtg acagggcgag atcaatcaca |  |
| 2.34 | 634 | 635 | HS_B_ | ttaaaacagc ctgtgggttg tacccaccca cagggcccac tgggcgctag cactctggta  ttgcggtacc tttgtgcgcc tgttttaccc accctccccc aacgtaactt agaagcctga  catatacggt cacaagccag cccagtacgc caactgggta ccgaccaagc acttctgtta  ccccggactg agtatcaata agctgccaaa gcggctgaag gagaaaacgt tcgttacccg  gccaattact tcgagaaacc cagtaacacc atgaaagttg cgtagcgttt cattcagcac  aatcccagtg tagatcaggt cgatgagtca ccgcattccc cacaggcgac tgtggcggtg  gctgcgttgg cggcctgccc atggggcaaa cccatgggac gcttcaatac tgacatggtg  cgaagagtct attgagctaa ttggtagtcc tccggcccct gaatgcggct aatcctaact  gcggagcagg cactcgcaga ccagcgagca gcttgtcgta atgggcaact ccgcagcgga  accgactact ttgggtgtcc gtgtttccta tttcctttac actggctgct tatggtgaca  attgagaaat tgttaccata tagctattgg agtgccatcc ggtgaccaat agagctatta  tatatctctt tgttgggttt ataccactta gcttgaaaga ggttaaaaca ttacaattca  ttgttaagtt gaatacagca aaatgggagc tcaagtatca acgcaaaaga ctggggcaca  tgagaccggg ctgaatgcta gcggcaattc catcattcac tacacaaatg ttaattatta  caaggatgcc gcatccaact cagccaatcg gcaggatttc actcaagacc cgggcaagtt  cacagaacca gtaaaagata tcatgattaa atcactacca gctctcaact cccccacagt  agaggagtgc ggatacagtg acagggcgag atcaatcaca |  |
| 2.36 | 634 | 635 | HS_B_ | ttaaaacagc ctgtgggttg tacccaccca cagggcccac tgggcgctag cactctggta  ttgcggtacc tttgtgcgcc tgttttaccc accctccccc aacgtaactt agaagcctga  catatacggt cacaagccag cccagtacgc caactgggta ccgaccaagc acttctgtta  ccccggactg agtatcaata agctgccaaa gcggctgaag gagaaaacgt tcgttacccg  gccaattact tcgagaaacc cagtaacacc atgaaagttg cgtagcgttt cattcagcac  aatcccagtg tagatcaggt cgatgagtca ccgcattccc cacaggcgac tgtggcggtg  gctgcgttgg cggcctgccc atggggcaaa cccatgggac gcttcaatac tgacatggtg  cgaagagtct attgagctaa ttggtagtcc tccggcccct gaatgcggct aatcctaact  gcggagcagg cactcgcaga ccagcgagca gcttgtcgta atgggcaact ccgcagcgga  accgactact ttgggtgtcc gtgtttccta tttcctttac actggctgct tatggtgaca  attgagaaat tgttaccata tagctattgg agtgccatcc ggtgaccaat agagctatta  tatatctctt tgttgggttt ataccactta gcttgaaaga ggttaaaaca ttacaattca  ttgttaagtt gaatacagca aaatgggagc tcaagtatca acgcaaaaga ctggggcaca  tgagaccggg ctgaatgcta gcggcaattc catcattcac tacacaaatg ttaattatta  caaggatgcc gcatccaact cagccaatcg gcaggatttc actcaagacc cgggcaagtt  cacagaacca gtaaaagata tcatgattaa atcactacca gctctcaact cccccacagt  agaggagtgc ggatacagtg acagggcgag atcaatcaca |  |
| 2.37 | 575 | 576 | NHS | ttaaaacagc ctgtgggttg tacccaccca cagggcccac tgggcgctag cactctggta  ttgcggtacc tttgtgcgcc tgttttaccc accctccccc aacgtaactt agaagcctga  catatacggt cacaagccag cccagtacgc caactgggta ccgaccaagc acttctgtta  ccccggactg agtatcaata agctgccaaa gcggctgaag gagaaaacgt tcgttacccg  gccaattact tcgagaaacc cagtaacacc atgaaagttg cgtagcgttt cattcagcac  aatcccagtg tagatcaggt cgatgagtca ccgcattccc cacaggcgac tgtggcggtg  gctgcgttgg cggcctgccc atggggcaaa cccatgggac gcttcaatac tgacatggtg  cgaagagtct attgagctaa ttggtagtcc tccggcccct gaatgcggct aatcctaact  gcggagcagg cactcgcaga ccagcgagca gcttgtcgta atgggcaact ccgcagcgga  accgactact ttgggtgtcc gtgtttccta tttccctata ctggctgctt atggtgacaa  ttgagagatt gttaccatat agctattgga ttggccatcc ggtgaccaat agagctatta  tatatctctt tgttgggttt ataccactta gcttgaaaga ggttaaaaca ttacaattca  ttgttaagtt gaatacagca aaatgggagc tcaagtatca acgcaaaaga ctggggcaca  tgagaccggg ctgaatgcta gcggcaattc catcattcac tacacaaatg ttaattatta  caaggatgcc gcatccaact cagccaatcg gcaggatttc actcaagacc cgggcaagtt  cacagaacca gtaaaagata tcatgattaa atcactacca gctctcaact cccccacagt  agaggagtgc ggatacagtg acagggcgag atcaatcaca |  |
| 2.38 | 133 | 134 | HS_A_ | ttaaaacagc ctgtgggttg tacccaccca cagggcccac tgggcgctag cactctggta  ttgcggtacc tttgtgcgcc tgttttaccc accctccccc aacgtaactt agaagcctga  catatacggt caccagtcag cgtggcacac cagccacgtt ttgatcaagc acttctgtta  ccccggactg agtatcaata gactgctcac gcggttgaag gagaaagcgt tcgttatccg  gccaactact tcgaaaaacc tagtaacacc gtggaagttg cagagtgttt cgctcagcac  taccccagtg tagatcaggt cgatgagtca ccgcattccc cacgggcgac cgtggcggtg  gctgcgttgg cggcctgccc atggggaaac ccatgggacg ctctaataca gacatggtgc  gaagagtcta ttgagctagt tggtagtcct ccggcccctg aatgcggcta atcctaactg  cggagcacac accctcaagc cagagggcag tgtgtcgtaa cgggcaactc tgcagcggaa  ccgactactt tgggtgtccg tgtttcattt tattcctata ctggctgctt atggtgacaa  ttgagagatt gttaccatat agctattgga ttggccatcc ggtgaccaat agagctatta  tatatctctt tgttgggttt ataccactta gcttgaaaga ggttaaaaca ttacaattca  ttgttaagtt gaatacagca aaatgggagc tcaagtatca acgcaaaaga ctggggcaca  tgagaccggg ctgaatgcta gcggcaattc catcattcac tacacaaatg ttaattatta  caaggatgcc gcatccaact cagccaatcg gcaggatttc actcaagacc cgggcaagtt  cacagaacca gtaaaagata tcatgattaa atcactacca gctctcaact cccccacagt  agaggagtgc ggatacagtg acagggcgag atcaatcaca |  |
| 3.14 | 872 | 873 | NHS | ttaaaacagc ctgtgggttg tacccaccca cagggcccac tgggcgctag cactctggta  ttgcggtacc tttgtgcgcc tgttttaccc accctccccc aacgtaactt agaagcctga  catatacggt cacaagccag cccagtacgc caactgggta ccgaccaagc acttctgtta  ccccggactg agtatcaata agctgccaaa gcggctgaag gagaaaacgt tcgttacccg  gccaattact tcgagaaacc cagtaacacc atgaaagttg cgtagcgttt cattcagcac  aatcccagtg tagatcaggt cgatgagtca ccgcattccc cacaggcgac tgtggcggtg  gctgcgttgg cggcctgccc atggggcaaa cccatgggac gcttcaatac tgacatggtg  cgaagagtct attgagctaa ttggtagtcc tccggcccct gaatgcggct aatcctaact  gcggagcagg cactcgcaga ccagcgagca gcttgtcgta atgggcaact ccgcagcgga  accgactact ttgggtgtcc gtgtttccta tttcctttac actggctgct tatggtgaca  attgagaaat tgttaccata tagctattgg agtggccatc cggtgactaa cagagcaata  atatatttgt ttgttggctt tgtgccactt aatttgaagg ttgttaagac gctcaatctg  atactacttc taaataaagc aaaatgggag ctcaagtctc aacacagaaa acaggagctc  acgaaaccgc attaaacgcg cagggaaact cggtacttca ctacaccaac ataaattact  acaaggatgc ggcttccaac tcagcaaata ggaggatttc actcaagacc cgggcaagtt  cacagaacca gtaaaagata tcatgattaa atcactacca gctctcaact cccccacagt  agaggagtgc ggatacagtg acagggcgag atcaatcaca |  |
| 3.41 | 872 | 873 | NHS | ttaaaacagc ctgtgggttg tacccaccca cagggcccac tgggcgctag cactctggta  ttgcggtacc tttgtgcgcc tgttttaccc accctccccc aacgtaactt agaagcctga  catatacggt cacaagccag cccagtacgc caactgggta ccgaccaagc acttctgtta  ccccggactg agtatcaata agctgccaaa gcggctgaag gagaaaacgt tcgttacccg  gccaattact tcgagaaacc cagtaacacc atgaaagttg cgtagcgttt cattcagcac  aatcccagtg tagatcaggt cgatgagtca ccgcattccc cacaggcgac tgtggcggtg  gctgcgttgg cggcctgccc atggggcaaa cccatgggac gcttcaatac tgacatggtg  cgaagagtct attgagctaa ttggtagtcc tccggcccct gaatgcggct aatcctaact  gcggagcagg cactcgcaga ccagcgagca gcttgtcgta atgggcaact ccgcagcgga  accgactact ttgggtgtcc gtgtttccta tttcctttac actggctgct tatggtgaca  attgagaaat tgttaccata tagctattgg agtggccatc cggtgactaa cagagcaata  atatatttgt ttgttggctt tgtgccactt aatttgaagg ttgttaagac gctcaatctg  atactacttc taaataaagc aaaatgggag ctcaagtctc aacacagaaa acaggagctc  acgaaaccgc attaaacgcg cagggaaact cggtacttca ctacaccaac ataaattact  acaaggatgc ggcttccaac tcagcaaata ggaggatttc actcaagacc cgggcaagtt  cacagaacca gtaaaagata tcatgattaa atcactacca gctctcaact cccccacagt  agaggagtgc ggatacagtg acagggcgag atcaatcaca |  |
| 3.48 | 872 | 873 | NHS | ttaaaacagc ctgtgggttg tacccaccca cagggcccac tgggcgctag cactctggta  ttgcggtacc tttgtgcgcc tgttttaccc accctccccc aacgtaactt agaagcctga  catatacggt cacaagccag cccagtacgc caactgggta ccgaccaagc acttctgtta  ccccggactg agtatcaata agctgccaaa gcggctgaag gagaaaacgt tcgttacccg  gccaattact tcgagaaacc cagtaacacc atgaaagttg cgtagcgttt cattcagcac  aatcccagtg tagatcaggt cgatgagtca ccgcattccc cacaggcgac tgtggcggtg  gctgcgttgg cggcctgccc atggggcaaa cccatgggac gcttcaatac tgacatggtg  cgaagagtct attgagctaa ttggtagtcc tccggcccct gaatgcggct aatcctaact  gcggagcagg cactcgcaga ccagcgagca gcttgtcgta atgggcaact ccgcagcgga  accgactact ttgggtgtcc gtgtttccta tttcctttac actggctgct tatggtgaca  attgagaaat tgttaccata tagctattgg agtggccatc cggtgactaa cagagcaata  atatatttgt ttgttggctt tgtgccactt aatttgaagg ttgttaagac gctcaatctg  atactacttc taaataaagc aaaatgggag ctcaagtctc aacacagaaa acaggagctc  acgaaaccgc attaaacgcg cagggaaact cggtacttca ctacaccaac ataaattact  acaaggatgc ggcttccaac tcagcaaata ggaggatttc actcaagacc cgggcaagtt  cacagaacca gtaaaagata tcatgattaa atcactacca gctctcaact cccccacagt  agaggagtgc ggatacagtg acagggcgag atcaatcaca |  |

| Non-homologous recombinants with deletions (Rec NH-) | | | | |
| --- | --- | --- | --- | --- |
|  | CV-B6/Schmitt | CV-B3/28 | Hot Spot | Sequence |
| 1.3 | 696 | 738 | HS_B_ | ttaaaacagc ctgtgggttg tacccaccca cagggcccac tgggcgctag cactctggta  ttgcggtacc tttgtgcgcc tgttttaccc accctccccc aacgtaactt agaagcctga  catatacggt cacaagccag cccagtacgc caactgggta ccgaccaagc acttctgtta  ccccggactg agtatcaata agctgccaaa gcggctgaag gagaaaacgt tcgttacccg  gccaattact tcgagaaacc cagtaacacc atgaaagttg cgtagcgttt cattcagcac  aatcccagtg tagatcaggt cgatgagtca ccgcattccc cacaggcgac tgtggcggtg  gctgcgttgg cggcctgccc atggggcaaa cccatgggac gcttcaatac tgacatggtg  cgaagagtct attgagctaa ttggtagtcc tccggcccct gaatgcggct aatcctaact  gcggagcagg cactcgcaga ccagcgagca gcttgtcgta atgggcaact ccgcagcgga  accgactact ttgggtgtcc gtgtttccta tttcctttac actggctgct tatggtgaca  attgagaaat tgttaccata tagctattgg agtggccatc cggtgactaa cagagcaata  atatatttgt ttgttggctt tgtgccactt aatttg  gca aaatgggagc tcaagtatca acgcaaaaga ctggggcaca  tgagaccggg ctgaatgcta gcggcaattc catcattcac tacacaaatg ttaattatta  caaggatgcc gcatccaact cagccaatcg gcaggatttc actcaagacc cgggcaagtt  cacagaacca gtaaaagata tcatgattaa atcactacca gctctcaact cccccacagt  agaggagtgc ggatacagtg acagggcgag atcaatcaca |
| 1.5 | 697 | 738 | HS_B_ | ttaaaacagc ctgtgggttg tacccaccca cagggcccac tgggcgctag cactctggta  ttgcggtacc tttgtgcgcc tgttttaccc accctccccc aacgtaactt agaagcctga  catatacggt cacaagccag cccagtacgc caactgggta ccgaccaagc acttctgtta  ccccggactg agtatcaata agctgccaaa gcggctgaag gagaaaacgt tcgttacccg  gccaattact tcgagaaacc cagtaacacc atgaaagttg cgtagcgttt cattcagcac  aatcccagtg tagatcaggt cgatgagtca ccgcattccc cacaggcgac tgtggcggtg  gctgcgttgg cggcctgccc atggggcaaa cccatgggac gcttcaatac tgacatggtg  cgaagagtct attgagctaa ttggtagtcc tccggcccct gaatgcggct aatcctaact  gcggagcagg cactcgcaga ccagcgagca gcttgtcgta atgggcaact ccgcagcgga  accgactact ttgggtgtcc gtgtttccta tttcctttac actggctgct tatggtgaca  attgagaaat tgttaccata tagctattgg agtggccatc cggtgactaa cagagcaata  atatatttgt ttgttggctt tgtgccactt aatttga  gca aaatgggagc tcaagtatca acgcaaaaga ctggggcaca  tgagaccggg ctgaatgcta gcggcaattc catcattcac tacacaaatg ttaattatta  caaggatgcc gcatccaact cagccaatcg gcaggatttc actcaagacc cgggcaagtt  cacagaacca gtaaaagata tcatgattaa atcactacca gctctcaact cccccacagt  agaggagtgc ggatacagtg acagggcgag atcaatcaca |
| 1.12 | 644 | 702 | HS_B_ | ttaaaacagc ctgtgggttg tacccaccca cagggcccac tgggcgctag cactctggta  ttgcggtacc tttgtgcgcc tgttttaccc accctccccc aacgtaactt agaagcctga  catatacggt cacaagccag cccagtacgc caactgggta ccgaccaagc acttctgtta  ccccggactg agtatcaata agctgccaaa gcggctgaag gagaaaacgt tcgttacccg  gccaattact tcgagaaacc cagtaacacc atgaaagttg cgtagcgttt cattcagcac  aatcccagtg tagatcaggt cgatgagtca ccgcattccc cacaggcgac tgtggcggtg  gctgcgttgg cggcctgccc atggggcaaa cccatgggac gcttcaatac tgacatggtg  cgaagagtct attgagctaa ttggtagtcc tccggcccct gaatgcggct aatcctaact  gcggagcagg cactcgcaga ccagcgagca gcttgtcgta atgggcaact ccgcagcgga  accgactact ttgggtgtcc gtgtttccta tttcctttac actggctgct tatggtgaca  attgagaaat tgttaccata tagctattgg agtggccatc cggt  ttaaaaca ttacaattca  ttgttaagtt gaatacagca aaatgggagc tcaagtatca acgcaaaaga ctggggcaca  tgagaccggg ctgaatgcta gcggcaattc catcattcac tacacaaatg ttaattatta  caaggatgcc gcatccaact cagccaatcg gcaggatttc actcaagacc cgggcaagtt  cacagaacca gtaaaagata tcatgattaa atcactacca gctctcaact cccccacagt  agaggagtgc ggatacagtg acagggcgag atcaatcaca |
| 1.13 | 518 | 632 | NHS | ttaaaacagc ctgtgggttg tacccaccca cagggcccac tgggcgctag cactctggta  ttgcggtacc tttgtgcgcc tgttttaccc accctccccc aacgtaactt agaagcctga  catatacggt cacaagccag cccagtacgc caactgggta ccgaccaagc acttctgtta  ccccggactg agtatcaata agctgccaaa gcggctgaag gagaaaacgt tcgttacccg  gccaattact tcgagaaacc cagtaacacc atgaaagttg cgtagcgttt cattcagcac  aatcccagtg tagatcaggt cgatgagtca ccgcattccc cacaggcgac tgtggcggtg  gctgcgttgg cggcctgccc atggggcaaa cccatgggac gcttcaatac tgacatggtg  cgaagagtct attgagctaa ttggtagtcc tccggcccct gaatgcggct aatcctaact  gcggagcagg cactcgcaga ccagcgagca gcttgtcg  tggccatcc ggtgaccaat agagctatta  tatatctctt tgttgggttt ataccactta gcttgaaaga ggttaaaaca ttacaattca  ttgttaagtt gaatacagca aaatgggagc tcaagtatca acgcaaaaga ctggggcaca  tgagaccggg ctgaatgcta gcggcaattc catcattcac tacacaaatg ttaattatta  caaggatgcc gcatccaact cagccaatcg gcaggatttc actcaagacc cgggcaagtt  cacagaacca gtaaaagata tcatgattaa atcactacca gctctcaact cccccacagt  agaggagtgc ggatacagtg acagggcgag atcaatcaca |
| 1.13bis | 623 | 635 | HS_B_ | ttaaaacagc ctgtgggttg tacccaccca cagggcccac tgggcgctag cactctggta  ttgcggtacc tttgtgcgcc tgttttaccc accctccccc aacgtaactt agaagcctga  catatacggt cacaagccag cccagtacgc caactgggta ccgaccaagc acttctgtta  ccccggactg agtatcaata agctgccaaa gcggctgaag gagaaaacgt tcgttacccg  gccaattact tcgagaaacc cagtaacacc atgaaagttg cgtagcgttt cattcagcac  aatcccagtg tagatcaggt cgatgagtca ccgcattccc cacaggcgac tgtggcggtg  gctgcgttgg cggcctgccc atggggcaaa cccatgggac gcttcaatac tgacatggtg  cgaagagtct attgagctaa ttggtagtcc tccggcccct gaatgcggct aatcctaact  gcggagcagg cactcgcaga ccagcgagca gcttgtcgta atgggcaact ccgcagcgga  accgactact ttgggtgtcc gtgtttccta tttcctttac actggctgct tatggtgaca  attgagaaat tgttaccata tag catcc ggtgaccaat agagctatta  tatatctctt tgttgggttt ataccactta gcttgaaaga ggttaaaaca ttacaattca  ttgttaagtt gaatacagca aaatgggagc tcaagtatca acgcaaaaga ctggggcaca  tgagaccggg ctgaatgcta gcggcaattc catcattcac tacacaaatg ttaattatta  caaggatgcc gcatccaact cagccaatcg gcaggatttc actcaagacc cgggcaagtt  cacagaacca gtaaaagata tcatgattaa atcactacca gctctcaact cccccacagt  agaggagtgc ggatacagtg acagggcgag atcaatcaca |
| 1.21 | 623 | 635 | HS_B_ | ttaaaacagc ctgtgggttg tacccaccca cagggcccac tgggcgctag cactctggta  ttgcggtacc tttgtgcgcc tgttttaccc accctccccc aacgtaactt agaagcctga  catatacggt cacaagccag cccagtacgc caactgggta ccgaccaagc acttctgtta  ccccggactg agtatcaata agctgccaaa gcggctgaag gagaaaacgt tcgttacccg  gccaattact tcgagaaacc cagtaacacc atgaaagttg cgtagcgttt cattcagcac  aatcccagtg tagatcaggt cgatgagtca ccgcattccc cacaggcgac tgtggcggtg  gctgcgttgg cggcctgccc atggggcaaa cccatgggac gcttcaatac tgacatggtg  cgaagagtct attgagctaa ttggtagtcc tccggcccct gaatgcggct aatcctaact  gcggagcagg cactcgcaga ccagcgagca gcttgtcgta atgggcaact ccgcagcgga  accgactact ttgggtgtcc gtgtttccta tttcctttac actggctgct tatggtgaca  attgagaaat tgttaccata tag catcc ggtgaccaat agagctatta  tatatctctt tgttgggttt ataccactta gcttgaaaga ggttaaaaca ttacaattca  ttgttaagtt gaatacagca aaatgggagc tcaagtatca acgcaaaaga ctggggcaca  tgagaccggg ctgaatgcta gcggcaattc catcattcac tacacaaatg ttaattatta  caaggatgcc gcatccaact cagccaatcg gcaggatttc actcaagacc cgggcaagtt  cacagaacca gtaaaagata tcatgattaa atcactacca gctctcaact cccccacagt  agaggagtgc ggatacagtg acagggcgag atcaatcaca |
| 1.22 | 604 | 638 | NHS | ttaaaacagc ctgtgggttg tacccaccca cagggcccac tgggcgctag cactctggta  ttgcggtacc tttgtgcgcc tgttttaccc accctccccc aacgtaactt agaagcctga  catatacggt cacaagccag cccagtacgc caactgggta ccgaccaagc acttctgtta  ccccggactg agtatcaata agctgccaaa gcggctgaag gagaaaacgt tcgttacccg  gccaattact tcgagaaacc cagtaacacc atgaaagttg cgtagcgttt cattcagcac  aatcccagtg tagatcaggt cgatgagtca ccgcattccc cacaggcgac tgtggcggtg  gctgcgttgg cggcctgccc atggggcaaa cccatgggac gcttcaatac tgacatggtg  cgaagagtct attgagctaa ttggtagtcc tccggcccct gaatgcggct aatcctaact  gcggagcagg cactcgcaga ccagcgagca gcttgtcgta atgggcaact ccgcagcgga  accgactact ttgggtgtcc gtgtttccta tttcctttac actggctgct tatggtgaca  attg tcc ggtgaccaat agagctatta  tatatctctt tgttgggttt ataccactta gcttgaaaga ggttaaaaca ttacaattca  ttgttaagtt gaatacagca aaatgggagc tcaagtatca acgcaaaaga ctggggcaca  tgagaccggg ctgaatgcta gcggcaattc catcattcac tacacaaatg ttaattatta  caaggatgcc gcatccaact cagccaatcg gcaggatttc actcaagacc cgggcaagtt  cacagaacca gtaaaagata tcatgattaa atcactacca gctctcaact cccccacagt  agaggagtgc ggatacagtg acagggcgag atcaatcaca |
| 1.23 | 665 | 748 | HS_B_ | ttaaaacagc ctgtgggttg tacccaccca cagggcccac tgggcgctag cactctggta  ttgcggtacc tttgtgcgcc tgttttaccc accctccccc aacgtaactt agaagcctga  catatacggt cacaagccag cccagtacgc caactgggta ccgaccaagc acttctgtta  ccccggactg agtatcaata agctgccaaa gcggctgaag gagaaaacgt tcgttacccg  gccaattact tcgagaaacc cagtaacacc atgaaagttg cgtagcgttt cattcagcac  aatcccagtg tagatcaggt cgatgagtca ccgcattccc cacaggcgac tgtggcggtg  gctgcgttgg cggcctgccc atggggcaaa cccatgggac gcttcaatac tgacatggtg  cgaagagtct attgagctaa ttggtagtcc tccggcccct gaatgcggct aatcctaact  gcggagcagg cactcgcaga ccagcgagca gcttgtcgta atgggcaact ccgcagcgga  accgactact ttgggtgtcc gtgtttccta tttcctttac actggctgct tatggtgaca  attgagaaat tgttaccata tagctattgg agtggccatc cggtgactaa cagagcaata  atata  agc tcaagtatca acgcaaaaga ctggggcaca  tgagaccggg ctgaatgcta gcggcaattc catcattcac tacacaaatg ttaattatta  caaggatgcc gcatccaact cagccaatcg gcaggatttc actcaagacc cgggcaagtt  cacagaacca gtaaaagata tcatgattaa atcactacca gctctcaact cccccacagt  agaggagtgc ggatacagtg acagggcgag atcaatcaca |
| 1.26 | 691 | 729 | HS_B_ | ttaaaacagc ctgtgggttg tacccaccca cagggcccac tgggcgctag cactctggta  ttgcggtacc tttgtgcgcc tgttttaccc accctccccc aacgtaactt agaagcctga  catatacggt cacaagccag cccagtacgc caactgggta ccgaccaagc acttctgtta  ccccggactg agtatcaata agctgccaaa gcggctgaag gagaaaacgt tcgttacccg  gccaattact tcgagaaacc cagtaacacc atgaaagttg cgtagcgttt cattcagcac  aatcccagtg tagatcaggt cgatgagtca ccgcattccc cacaggcgac tgtggcggtg  gctgcgttgg cggcctgccc atggggcaaa cccatgggac gcttcaatac tgacatggtg  cgaagagtct attgagctaa ttggtagtcc tccggcccct gaatgcggct aatcctaact  gcggagcagg cactcgcaga ccagcgagca gcttgtcgta atgggcaact ccgcagcgga  accgactact ttgggtgtcc gtgtttccta tttcctttac actggctgct tatggtgaca  attgagaaat tgttaccata tagctattgg agtggccatc cggtgactaa cagagcaata  atatatttgt ttgttggctt tgtgccactt a  tt gaatacagca aaatgggagc tcaagtatca acgcaaaaga ctggggcaca  tgagaccggg ctgaatgcta gcggcaattc catcattcac tacacaaatg ttaattatta  caaggatgcc gcatccaact cagccaatcg gcaggatttc actcaagacc cgggcaagtt  cacagaacca gtaaaagata tcatgattaa atcactacca gctctcaact cccccacagt  agaggagtgc ggatacagtg acagggcgag atcaatcaca |
| 1.36 | 647 | 709 | HS_B_ | ttaaaacagc ctgtgggttg tacccaccca cagggcccac tgggcgctag cactctggta  ttgcggtacc tttgtgcgcc tgttttaccc accctccccc aacgtaactt agaagcctga  catatacggt cacaagccag cccagtacgc caactgggta ccgaccaagc acttctgtta  ccccggactg agtatcaata agctgccaaa gcggctgaag gagaaaacgt tcgttacccg  gccaattact tcgagaaacc cagtaacacc atgaaagttg cgtagcgttt cattcagcac  aatcccagtg tagatcaggt cgatgagtca ccgcattccc cacaggcgac tgtggcggtg  gctgcgttgg cggcctgccc atggggcaaa cccatgggac gcttcaatac tgacatggtg  cgaagagtct attgagctaa ttggtagtcc tccggcccct gaatgcggct aatcctaact  gcggagcagg cactcgcaga ccagcgagca gcttgtcgta atgggcaact ccgcagcgga  accgactact ttgggtgtcc gtgtttccta tttcctttac actggctgct tatggtgaca  attgagaaat tgttaccata tagctattgg agtggccatc cggtgac  ca ttacaattca  ttgttaagtt gaatacagca aaatgggagc tcaagtatca acgcaaaaga ctggggcaca  tgagaccggg ctgaatgcta gcggcaattc catcattcac tacacaaatg ttaattatta  caaggatgcc gcatccaact cagccaatcg gcaggatttc actcaagacc cgggcaagtt  cacagaacca gtaaaagata tcatgattaa atcactacca gctctcaact cccccacagt  agaggagtgc ggatacagtg acagggcgag atcaatcaca |
| 1.37 | 649 | 711 | HS_B_ | ttaaaacagc ctgtgggttg tacccaccca cagggcccac tgggcgctag cactctggta  ttgcggtacc tttgtgcgcc tgttttaccc accctccccc aacgtaactt agaagcctga  catatacggt cacaagccag cccagtacgc caactgggta ccgaccaagc acttctgtta  ccccggactg agtatcaata agctgccaaa gcggctgaag gagaaaacgt tcgttacccg  gccaattact tcgagaaacc cagtaacacc atgaaagttg cgtagcgttt cattcagcac  aatcccagtg tagatcaggt cgatgagtca ccgcattccc cacaggcgac tgtggcggtg  gctgcgttgg cggcctgccc atggggcaaa cccatgggac gcttcaatac tgacatggtg  cgaagagtct attgagctaa ttggtagtcc tccggcccct gaatgcggct aatcctaact  gcggagcagg cactcgcaga ccagcgagca gcttgtcgta atgggcaact ccgcagcgga  accgactact ttgggtgtcc gtgtttccta tttcctttac actggctgct tatggtgaca  attgagaaat tgttaccata tagctattgg agtggccatc cggtgacta  ttacaattca  ttgttaagtt gaatacagca aaatgggagc tcaagtatca acgcaaaaga ctggggcaca  tgagaccggg ctgaatgcta gcggcaattc catcattcac tacacaaatg ttaattatta  caaggatgcc gcatccaact cagccaatcg gcaggatttc actcaagacc cgggcaagtt  cacagaacca gtaaaagata tcatgattaa atcactacca gctctcaact cccccacagt  agaggagtgc ggatacagtg acagggcgag atcaatcaca |
| 1.50 | 741 | 746 | HS_B_ | ttaaaacagc ctgtgggttg tacccaccca cagggcccac tgggcgctag cactctggta  ttgcggtacc tttgtgcgcc tgttttaccc accctccccc aacgtaactt agaagcctga  catatacggt cacaagccag cccagtacgc caactgggta ccgaccaagc acttctgtta  ccccggactg agtatcaata agctgccaaa gcggctgaag gagaaaacgt tcgttacccg  gccaattact tcgagaaacc cagtaacacc atgaaagttg cgtagcgttt cattcagcac  aatcccagtg tagatcaggt cgatgagtca ccgcattccc cacaggcgac tgtggcggtg  gctgcgttgg cggcctgccc atggggcaaa cccatgggac gcttcaatac tgacatggtg  cgaagagtct attgagctaa ttggtagtcc tccggcccct gaatgcggct aatcctaact  gcggagcagg cactcgcaga ccagcgagca gcttgtcgta atgggcaact ccgcagcgga  accgactact ttgggtgtcc gtgtttccta tttcctttac actggctgct tatggtgaca  attgagaaat tgttaccata tagctattgg agtggccatc cggtgactaa cagagcaata  atatatttgt ttgttggctt tgtgccactt aatttgaagg ttgttaagac gctcaatctg  atactacttc taaataaagc a ggagc tcaagtatca acgcaaaaga ctggggcaca  tgagaccggg ctgaatgcta gcggcaattc catcattcac tacacaaatg ttaattatta  caaggatgcc gcatccaact cagccaatcg gcaggatttc actcaagacc cgggcaagtt  cacagaacca gtaaaagata tcatgattaa atcactacca gctctcaact cccccacagt  agaggagtgc ggatacagtg acagggcgag atcaatcaca |
| 1.54 | 611 | 690 | NHS | ttaaaacagc ctgtgggttg tacccaccca cagggcccac tgggcgctag cactctggta  ttgcggtacc tttgtgcgcc tgttttaccc accctccccc aacgtaactt agaagcctga  catatacggt cacaagccag cccagtacgc caactgggta ccgaccaagc acttctgtta  ccccggactg agtatcaata agctgccaaa gcggctgaag gagaaaacgt tcgttacccg  gccaattact tcgagaaacc cagtaacacc atgaaagttg cgtagcgttt cattcagcac  aatcccagtg tagatcaggt cgatgagtca ccgcattccc cacaggcgac tgtggcggtg  gctgcgttgg cggcctgccc atggggcaaa cccatgggac gcttcaatac tgacatggtg  cgaagagtct attgagctaa ttggtagtcc tccggcccct gaatgcggct aatcctaact  gcggagcagg cactcgcaga ccagcgagca gcttgtcgta atgggcaact ccgcagcgga  accgactact ttgggtgtcc gtgtttccta tttcctttac actggctgct tatggtgaca  attgagaaat t a gcttgaaaga ggttaaaaca ttacaattca  ttgttaagtt gaatacagca aaatgggagc tcaagtatca acgcaaaaga ctggggcaca  tgagaccggg ctgaatgcta gcggcaattc catcattcac tacacaaatg ttaattatta  caaggatgcc gcatccaact cagccaatcg gcaggatttc actcaagacc cgggcaagtt  cacagaacca gtaaaagata tcatgattaa atcactacca gctctcaact cccccacagt  agaggagtgc ggatacagtg acagggcgag atcaatcaca |
| 1.58 | 668 | 688 | HS_B_ | ttaaaacagc ctgtgggttg tacccaccca cagggcccac tgggcgctag cactctggta  ttgcggtacc tttgtgcgcc tgttttaccc accctccccc aacgtaactt agaagcctga  catatacggt cacaagccag cccagtacgc caactgggta ccgaccaagc acttctgtta  ccccggactg agtatcaata agctgccaaa gcggctgaag gagaaaacgt tcgttacccg  gccaattact tcgagaaacc cagtaacacc atgaaagttg cgtagcgttt cattcagcac  aatcccagtg tagatcaggt cgatgagtca ccgcattccc cacaggcgac tgtggcggtg  gctgcgttgg cggcctgccc atggggcaaa cccatgggac gcttcaatac tgacatggtg  cgaagagtct attgagctaa ttggtagtcc tccggcccct gaatgcggct aatcctaact  gcggagcagg cactcgcaga ccagcgagca gcttgtcgta atgggcaact ccgcagcgga  accgactact ttgggtgtcc gtgtttccta tttcctttac actggctgct tatggtgaca  attgagaaat tgttaccata tagctattgg agtggccatc cggtgactaa cagagcaata  atatattt tta gcttgaaaga ggttaaaaca ttacaattca  ttgttaagtt gaatacagca aaatgggagc tcaagtatca acgcaaaaga ctggggcaca  tgagaccggg ctgaatgcta gcggcaattc catcattcac tacacaaatg ttaattatta  caaggatgcc gcatccaact cagccaatcg gcaggatttc actcaagacc cgggcaagtt  cacagaacca gtaaaagata tcatgattaa atcactacca gctctcaact cccccacagt  agaggagtgc ggatacagtg acagggcgag atcaatcaca |
| 1.59 | 624 | 685 | HS_B_ | ttaaaacagc ctgtgggttg tacccaccca cagggcccac tgggcgctag cactctggta  ttgcggtacc tttgtgcgcc tgttttaccc accctccccc aacgtaactt agaagcctga  catatacggt cacaagccag cccagtacgc caactgggta ccgaccaagc acttctgtta  ccccggactg agtatcaata agctgccaaa gcggctgaag gagaaaacgt tcgttacccg  gccaattact tcgagaaacc cagtaacacc atgaaagttg cgtagcgttt cattcagcac  aatcccagtg tagatcaggt cgatgagtca ccgcattccc cacaggcgac tgtggcggtg  gctgcgttgg cggcctgccc atggggcaaa cccatgggac gcttcaatac tgacatggtg  cgaagagtct attgagctaa ttggtagtcc tccggcccct gaatgcggct aatcctaact  gcggagcagg cactcgcaga ccagcgagca gcttgtcgta atgggcaact ccgcagcgga  accgactact ttgggtgtcc gtgtttccta tttcctttac actggctgct tatggtgaca  attgagaaat tgttaccata tagc  actta gcttgaaaga ggttaaaaca ttacaattca  ttgttaagtt gaatacagca aaatgggagc tcaagtatca acgcaaaaga ctggggcaca  tgagaccggg ctgaatgcta gcggcaattc catcattcac tacacaaatg ttaattatta  caaggatgcc gcatccaact cagccaatcg gcaggatttc actcaagacc cgggcaagtt  cacagaacca gtaaaagata tcatgattaa atcactacca gctctcaact cccccacagt  agaggagtgc ggatacagtg acagggcgag atcaatcaca |
| 1.64 | 713 | 742 | HS_B_ | ttaaaacagc ctgtgggttg tacccaccca cagggcccac tgggcgctag cactctggta  ttgcggtacc tttgtgcgcc tgttttaccc accctccccc aacgtaactt agaagcctga  catatacggt cacaagccag cccagtacgc caactgggta ccgaccaagc acttctgtta  ccccggactg agtatcaata agctgccaaa gcggctgaag gagaaaacgt tcgttacccg  gccaattact tcgagaaacc cagtaacacc atgaaagttg cgtagcgttt cattcagcac  aatcccagtg tagatcaggt cgatgagtca ccgcattccc cacaggcgac tgtggcggtg  gctgcgttgg cggcctgccc atggggcaaa cccatgggac gcttcaatac tgacatggtg  cgaagagtct attgagctaa ttggtagtcc tccggcccct gaatgcggct aatcctaact  gcggagcagg cactcgcaga ccagcgagca gcttgtcgta atgggcaact ccgcagcgga  accgactact ttgggtgtcc gtgtttccta tttcctttac actggctgct tatggtgaca  attgagaaat tgttaccata tagctattgg agtggccatc cggtgactaa cagagcaata  atatatttgt ttgttggctt tgtgccactt aatttgaagg ttgttaagac gct  atgggagc tcaagtatca acgcaaaaga ctggggcaca  tgagaccggg ctgaatgcta gcggcaattc catcattcac tacacaaatg ttaattatta  caaggatgcc gcatccaact cagccaatcg gcaggatttc actcaagacc cgggcaagtt  cacagaacca gtaaaagata tcatgattaa atcactacca gctctcaact cccccacagt  agaggagtgc ggatacagtg acagggcgag atcaatcaca |
| 1.65 | 702 | 716 | HS_B_ | ttaaaacagc ctgtgggttg tacccaccca cagggcccac tgggcgctag cactctggta  ttgcggtacc tttgtgcgcc tgttttaccc accctccccc aacgtaactt agaagcctga  catatacggt cacaagccag cccagtacgc caactgggta ccgaccaagc acttctgtta  ccccggactg agtatcaata agctgccaaa gcggctgaag gagaaaacgt tcgttacccg  gccaattact tcgagaaacc cagtaacacc atgaaagttg cgtagcgttt cattcagcac  aatcccagtg tagatcaggt cgatgagtca ccgcattccc cacaggcgac tgtggcggtg  gctgcgttgg cggcctgccc atggggcaaa cccatgggac gcttcaatac tgacatggtg  cgaagagtct attgagctaa ttggtagtcc tccggcccct gaatgcggct aatcctaact  gcggagcagg cactcgcaga ccagcgagca gcttgtcgta atgggcaact ccgcagcgga  accgactact ttgggtgtcc gtgtttccta tttcctttac actggctgct tatggtgaca  attgagaaat tgttaccata tagctattgg agtggccatc cggtgactaa cagagcaata  atatatttgt ttgttggctt tgtgccactt aatttgaagg tt attca  ttgttaagtt gaatacagca aaatgggagc tcaagtatca acgcaaaaga ctggggcaca  tgagaccggg ctgaatgcta gcggcaattc catcattcac tacacaaatg ttaattatta  caaggatgcc gcatccaact cagccaatcg gcaggatttc actcaagacc cgggcaagtt  cacagaacca gtaaaagata tcatgattaa atcactacca gctctcaact cccccacagt  agaggagtgc ggatacagtg acagggcgag atcaatcaca |
| 2.5 | 629 | 705 | HS_B_ | ttaaaacagc ctgtgggttg tacccaccca cagggcccac tgggcgctag cactctggta  ttgcggtacc tttgtgcgcc tgttttaccc accctccccc aacgtaactt agaagcctga  catatacggt cacaagccag cccagtacgc caactgggta ccgaccaagc acttctgtta  ccccggactg agtatcaata agctgccaaa gcggctgaag gagaaaacgt tcgttacccg  gccaattact tcgagaaacc cagtaacacc atgaaagttg cgtagcgttt cattcagcac  aatcccagtg tagatcaggt cgatgagtca ccgcattccc cacaggcgac tgtggcggtg  gctgcgttgg cggcctgccc atggggcaaa cccatgggac gcttcaatac tgacatggtg  cgaagagtct attgagctaa ttggtagtcc tccggcccct gaatgcggct aatcctaact  gcggagcagg cactcgcaga ccagcgagca gcttgtcgta atgggcaact ccgcagcgga  accgactact ttgggtgtcc gtgtttccta tttcctttac actggctgct tatggtgaca  attgagaaat tgttaccata tagctattg  aaaca ttacaattca  ttgttaagtt gaatacagca aaatgggagc tcaagtatca acgcaaaaga ctggggcaca  tgagaccggg ctgaatgcta gcggcaattc catcattcac tacacaaatg ttaattatta  caaggatgcc gcatccaact cagccaatcg gcaggatttc actcaagacc cgggcaagtt  cacagaacca gtaaaagata tcatgattaa atcactacca gctctcaact cccccacagt  agaggagtgc ggatacagtg acagggcgag atcaatcaca |
| 2.6 | 694 | 770 | NHS | ttaaaacagc ctgtgggttg tacccaccca cagggcccac tgggcgctag cactctggta  ttgcggtacc tttgtgcgcc tgttttaccc accctccccc aacgtaactt agaagcctga  catatacggt cacaagccag cccagtacgc caactgggta ccgaccaagc acttctgtta  ccccggactg agtatcaata agctgccaaa gcggctgaag gagaaaacgt tcgttacccg  gccaattact tcgagaaacc cagtaacacc atgaaagttg cgtagcgttt cattcagcac  aatcccagtg tagatcaggt cgatgagtca ccgcattccc cacaggcgac tgtggcggtg  gctgcgttgg cggcctgccc atggggcaaa cccatgggac gcttcaatac tgacatggtg  cgaagagtct attgagctaa ttggtagtcc tccggcccct gaatgcggct aatcctaact  gcggagcagg cactcgcaga ccagcgagca gcttgtcgta atgggcaact ccgcagcgga  accgactact ttgggtgtcc gtgtttccta tttcctttac actggctgct tatggtgaca  attgagaaat tgttaccata tagctattgg agtggccatc cggtgactaa cagagcaata  atatatttgt ttgttggctt tgtgccactt aatt  a ctggggcaca  tgagaccggg ctgaatgcta gcggcaattc catcattcac tacacaaatg ttaattatta  caaggatgcc gcatccaact cagccaatcg gcaggatttc actcaagacc cgggcaagtt  cacagaacca gtaaaagata tcatgattaa atcactacca gctctcaact cccccacagt  agaggagtgc ggatacagtg acagggcgag atcaatcaca |
| 2.7 | 598 | 684 | NHS | ttaaaacagc ctgtgggttg tacccaccca cagggcccac tgggcgctag cactctggta  ttgcggtacc tttgtgcgcc tgttttaccc accctccccc aacgtaactt agaagcctga  catatacggt cacaagccag cccagtacgc caactgggta ccgaccaagc acttctgtta  ccccggactg agtatcaata agctgccaaa gcggctgaag gagaaaacgt tcgttacccg  gccaattact tcgagaaacc cagtaacacc atgaaagttg cgtagcgttt cattcagcac  aatcccagtg tagatcaggt cgatgagtca ccgcattccc cacaggcgac tgtggcggtg  gctgcgttgg cggcctgccc atggggcaaa cccatgggac gcttcaatac tgacatggtg  cgaagagtct attgagctaa ttggtagtcc tccggcccct gaatgcggct aatcctaact  gcggagcagg cactcgcaga ccagcgagca gcttgtcgta atgggcaact ccgcagcgga  accgactact ttgggtgtcc gtgtttccta tttcctttac actggctgct tatggtga  cactta gcttgaaaga ggttaaaaca ttacaattca  ttgttaagtt gaatacagca aaatgggagc tcaagtatca acgcaaaaga ctggggcaca  tgagaccggg ctgaatgcta gcggcaattc catcattcac tacacaaatg ttaattatta  caaggatgcc gcatccaact cagccaatcg gcaggatttc actcaagacc cgggcaagtt  cacagaacca gtaaaagata tcatgattaa atcactacca gctctcaact cccccacagt  agaggagtgc ggatacagtg acagggcgag atcaatcaca |
| 2.12 | 649 | 716 | HS_B_ | ttaaaacagc ctgtgggttg tacccaccca cagggcccac tgggcgctag cactctggta  ttgcggtacc tttgtgcgcc tgttttaccc accctccccc aacgtaactt agaagcctga  catatacggt cacaagccag cccagtacgc caactgggta ccgaccaagc acttctgtta  ccccggactg agtatcaata agctgccaaa gcggctgaag gagaaaacgt tcgttacccg  gccaattact tcgagaaacc cagtaacacc atgaaagttg cgtagcgttt cattcagcac  aatcccagtg tagatcaggt cgatgagtca ccgcattccc cacaggcgac tgtggcggtg  gctgcgttgg cggcctgccc atggggcaaa cccatgggac gcttcaatac tgacatggtg  cgaagagtct attgagctaa ttggtagtcc tccggcccct gaatgcggct aatcctaact  gcggagcagg cactcgcaga ccagcgagca gcttgtcgta atgggcaact ccgcagcgga  accgactact ttgggtgtcc gtgtttccta tttcctttac actggctgct tatggtgaca  attgagaaat tgttaccata tagctattgg agtggccatc cggtgacta  attca  ttgttaagtt gaatacagca aaatgggagc tcaagtatca acgcaaaaga ctggggcaca  tgagaccggg ctgaatgcta gcggcaattc catcattcac tacacaaatg ttaattatta  caaggatgcc gcatccaact cagccaatcg gcaggatttc actcaagacc cgggcaagtt  cacagaacca gtaaaagata tcatgattaa atcactacca gctctcaact cccccacagt  agaggagtgc ggatacagtg acagggcgag atcaatcaca |
| 2.31 | 623 | 739 | HS_B_ | ttaaaacagc ctgtgggttg tacccaccca cagggcccac tgggcgctag cactctggta  ttgcggtacc tttgtgcgcc tgttttaccc accctccccc aacgtaactt agaagcctga  catatacggt cacaagccag cccagtacgc caactgggta ccgaccaagc acttctgtta  ccccggactg agtatcaata agctgccaaa gcggctgaag gagaaaacgt tcgttacccg  gccaattact tcgagaaacc cagtaacacc atgaaagttg cgtagcgttt cattcagcac  aatcccagtg tagatcaggt cgatgagtca ccgcattccc cacaggcgac tgtggcggtg  gctgcgttgg cggcctgccc atggggcaaa cccatgggac gcttcaatac tgacatggtg  cgaagagtct attgagctaa ttggtagtcc tccggcccct gaatgcggct aatcctaact  gcggagcagg cactcgcaga ccagcgagca gcttgtcgta atgggcaact ccgcagcgga  accgactact ttgggtgtcc gtgtttccta tttcctttac actggctgct tatggtgaca  attgagaaat tgttaccata tag  ca aaatgggagc tcaagtatca acgcaaaaga ctggggcaca  tgagaccggg ctgaatgcta gcggcaattc catcattcac tacacaaatg ttaattatta  caaggatgcc gcatccaact cagccaatcg gcaggatttc actcaagacc cgggcaagtt  cacagaacca gtaaaagata tcatgattaa atcactacca gctctcaact cccccacagt  agaggagtgc ggatacagtg acagggcgag atcaatcaca |
| 2.32 | 623 | 739 | HS_B_ | ttaaaacagc ctgtgggttg tacccaccca cagggcccac tgggcgctag cactctggta  ttgcggtacc tttgtgcgcc tgttttaccc accctccccc aacgtaactt agaagcctga  catatacggt cacaagccag cccagtacgc caactgggta ccgaccaagc acttctgtta  ccccggactg agtatcaata agctgccaaa gcggctgaag gagaaaacgt tcgttacccg  gccaattact tcgagaaacc cagtaacacc atgaaagttg cgtagcgttt cattcagcac  aatcccagtg tagatcaggt cgatgagtca ccgcattccc cacaggcgac tgtggcggtg  gctgcgttgg cggcctgccc atggggcaaa cccatgggac gcttcaatac tgacatggtg  cgaagagtct attgagctaa ttggtagtcc tccggcccct gaatgcggct aatcctaact  gcggagcagg cactcgcaga ccagcgagca gcttgtcgta atgggcaact ccgcagcgga  accgactact ttgggtgtcc gtgtttccta tttcctttac actggctgct tatggtgaca  attgagaaat tgttaccata tag  ca aaatgggagc tcaagtatca acgcaaaaga ctggggcaca  tgagaccggg ctgaatgcta gcggcaattc catcattcac tacacaaatg ttaattatta  caaggatgcc gcatccaact cagccaatcg gcaggatttc actcaagacc cgggcaagtt  cacagaacca gtaaaagata tcatgattaa atcactacca gctctcaact cccccacagt  agaggagtgc ggatacagtg acagggcgag atcaatcaca |
| 2.33 | 623 | 739 | HS_B_ | ttaaaacagc ctgtgggttg tacccaccca cagggcccac tgggcgctag cactctggta  ttgcggtacc tttgtgcgcc tgttttaccc accctccccc aacgtaactt agaagcctga  catatacggt cacaagccag cccagtacgc caactgggta ccgaccaagc acttctgtta  ccccggactg agtatcaata agctgccaaa gcggctgaag gagaaaacgt tcgttacccg  gccaattact tcgagaaacc cagtaacacc atgaaagttg cgtagcgttt cattcagcac  aatcccagtg tagatcaggt cgatgagtca ccgcattccc cacaggcgac tgtggcggtg  gctgcgttgg cggcctgccc atggggcaaa cccatgggac gcttcaatac tgacatggtg  cgaagagtct attgagctaa ttggtagtcc tccggcccct gaatgcggct aatcctaact  gcggagcagg cactcgcaga ccagcgagca gcttgtcgta atgggcaact ccgcagcgga  accgactact ttgggtgtcc gtgtttccta tttcctttac actggctgct tatggtgaca  attgagaaat tgttaccata tag  ca aaatgggagc tcaagtatca acgcaaaaga ctggggcaca  tgagaccggg ctgaatgcta gcggcaattc catcattcac tacacaaatg ttaattatta  caaggatgcc gcatccaact cagccaatcg gcaggatttc actcaagacc cgggcaagtt  cacagaacca gtaaaagata tcatgattaa atcactacca gctctcaact cccccacagt  agaggagtgc ggatacagtg acagggcgag atcaatcaca |

| Non-homologous recombinants with insertions (Rec NH+) | | | | |
| --- | --- | --- | --- | --- |
|  | CV-B6/Schmitt | CV-B3/28 | Hot Spot | Sequence |
| 1.14 | 216 | 132 | HS_A_ | ttaaaacagc ctgtgggttg tacccaccca cagggcccac tgggcgctag cactctggta  ttgcggtacc tttgtgcgcc tgttttaccc accctccccc aacgtaactt agaagcctga  catatacggt cacaagccag cccagtacgc caactgggta ccgaccaagc acttctgtta  ccccggactg agtatcaata agctgccaaa gcggct  aacagtcag cgtggcacac cagccacgtt ttgatcaagc acttctgtta  ccccggactg agtatcaata gactgctcac gcggttgaag gagaaagcgt tcgttatccg  gccaactact tcgaaaaacc tagtaacacc gtggaagttg cagagtgttt cgctcagcac  taccccagtg tagatcaggt cgatgagtca ccgcattccc cacgggcgac cgtggcggtg  gctgcgttgg cggcctgccc atggggaaac ccatgggacg ctctaataca gacatggtgc  gaagagtcta ttgagctagt tggtagtcct ccggcccctg aatgcggcta atcctaactg  cggagcacac accctcaagc cagagggcag tgtgtcgtaa cgggcaactc tgcagcggaa  ccgactactt tgggtgtccg tgtttcattt tattcctata ctggctgctt atggtgacaa  ttgagagatt gttaccatat agctattgga ttggccatcc ggtgaccaat agagctatta  tatatctctt tgttgggttt ataccactta gcttgaaaga ggttaaaaca ttacaattca  ttgttaagtt gaatacagca aaatgggagc tcaagtatca acgcaaaaga ctggggcaca  tgagaccggg ctgaatgcta gcggcaattc catcattcac tacacaaatg ttaattatta  caaggatgcc gcatccaact cagccaatcg gcaggatttc actcaagacc cgggcaagtt  cacagaacca gtaaaagata tcatgattaa atcactacca gctctcaact cccccacagt  agaggagtgc ggatacagtg acagggcgag atcaatcaca |
| 1.31 | 700 | 271 | NHS | ttaaaacagc ctgtgggttg tacccaccca cagggcccac tgggcgctag cactctggta  ttgcggtacc tttgtgcgcc tgttttaccc accctccccc aacgtaactt agaagcctga  catatacggt cacaagccag cccagtacgc caactgggta ccgaccaagc acttctgtta  ccccggactg agtatcaata agctgccaaa gcggctgaag gagaaaacgt tcgttacccg  gccaattact tcgagaaacc cagtaacacc atgaaagttg cgtagcgttt cattcagcac  aatcccagtg tagatcaggt cgatgagtca ccgcattccc cacaggcgac tgtggcggtg  gctgcgttgg cggcctgccc atggggcaaa cccatgggac gcttcaatac tgacatggtg  cgaagagtct attgagctaa ttggtagtcc tccggcccct gaatgcggct aatcctaact  gcggagcagg cactcgcaga ccagcgagca gcttgtcgta atgggcaact ccgcagcgga  accgactact ttgggtgtcc gtgtttccta tttcctttac actggctgct tatggtgaca  attgagaaat tgttaccata tagctattgg agtggccatc cggtgactaa cagagcaata  atatatttgt ttgttggctt tgtgccactt aatttgaagg  gtggaagttg cagagtgttt cgctcagcac  taccccagtg tagatcaggt cgatgagtca ccgcattccc cacgggcgac cgtggcggtg  gctgcgttgg cggcctgccc atggggaaac ccatgggacg ctctaataca gacatggtgc  gaagagtcta ttgagctagt tggtagtcct ccggcccctg aatgcggcta atcctaactg  cggagcacac accctcaagc cagagggcag tgtgtcgtaa cgggcaactc tgcagcggaa  ccgactactt tgggtgtccg tgtttcattt tattcctata ctggctgctt atggtgacaa  ttgagagatt gttaccatat agctattgga ttggccatcc ggtgaccaat agagctatta  tatatctctt tgttgggttt ataccactta gcttgaaaga ggttaaaaca ttacaattca  ttgttaagtt gaatacagca aaatgggagc tcaagtatca acgcaaaaga ctggggcaca  tgagaccggg ctgaatgcta gcggcaattc catcattcac tacacaaatg ttaattatta  caaggatgcc gcatccaact cagccaatcg gcaggatttc actcaagacc cgggcaagtt  cacagaacca gtaaaagata tcatgattaa atcactacca gctctcaact cccccacagt  agaggagtgc ggatacagtg acagggcgag atcaatcaca |
| 1.33 | 107 | 51 | HS_A_ | ttaaaacagc ctgtgggttg tacccaccca cagggcccac tgggcgctag cactctggta  ttgcggtacc tttgtgcgcc tgttttaccc accctccccc aacgtaa  cactctggta  tcacggtacc tttgtgcgcc tgttttatac cccctccccc aactgtaact tagaagtaac  acacaccgat caacagtcag cgtggcacac cagccacgtt ttgatcaagc acttctgtta  ccccggactg agtatcaata gactgctcac gcggttgaag gagaaagcgt tcgttatccg  gccaactact tcgaaaaacc tagtaacacc gtggaagttg cagagtgttt cgctcagcac  taccccagtg tagatcaggt cgatgagtca ccgcattccc cacgggcgac cgtggcggtg  gctgcgttgg cggcctgccc atggggaaac ccatgggacg ctctaataca gacatggtgc  gaagagtcta ttgagctagt tggtagtcct ccggcccctg aatgcggcta atcctaactg  cggagcacac accctcaagc cagagggcag tgtgtcgtaa cgggcaactc tgcagcggaa  ccgactactt tgggtgtccg tgtttcattt tattcctata ctggctgctt atggtgacaa  ttgagagatt gttaccatat agctattgga ttggccatcc ggtgaccaat agagctatta  tatatctctt tgttgggttt ataccactta gcttgaaaga ggttaaaaca ttacaattca  ttgttaagtt gaatacagca aaatgggagc tcaagtatca acgcaaaaga ctggggcaca  tgagaccggg ctgaatgcta gcggcaattc catcattcac tacacaaatg ttaattatta  caaggatgcc gcatccaact cagccaatcg gcaggatttc actcaagacc cgggcaagtt  cacagaacca gtaaaagata tcatgattaa atcactacca gctctcaact cccccacagt  agaggagtgc ggatacagtg acagggcgag atcaatcaca |
| 1.44 | 742 | 671 | HS_B_ | ttaaaacagc ctgtgggttg tacccaccca cagggcccac tgggcgctag cactctggta  ttgcggtacc tttgtgcgcc tgttttaccc accctccccc aacgtaactt agaagcctga  catatacggt cacaagccag cccagtacgc caactgggta ccgaccaagc acttctgtta  ccccggactg agtatcaata agctgccaaa gcggctgaag gagaaaacgt tcgttacccg  gccaattact tcgagaaacc cagtaacacc atgaaagttg cgtagcgttt cattcagcac  aatcccagtg tagatcaggt cgatgagtca ccgcattccc cacaggcgac tgtggcggtg  gctgcgttgg cggcctgccc atggggcaaa cccatgggac gcttcaatac tgacatggtg  cgaagagtct attgagctaa ttggtagtcc tccggcccct gaatgcggct aatcctaact  gcggagcagg cactcgcaga ccagcgagca gcttgtcgta atgggcaact ccgcagcgga  accgactact ttgggtgtcc gtgtttccta tttcctttac actggctgct tatggtgaca  attgagaaat tgttaccata tagctattgg agtggccatc cggtgactaa cagagcaata  atatatttgt ttgttggctt tgtgccactt aatttgaagg ttgttaagac gctcaatctg  atactacttc taaataaagc aa  tgttgggttt ataccactta gcttgaaaga ggttaaaaca ttacaattca  ttgttaagtt gaatacagca aaatgggagc tcaagtatca acgcaaaaga ctggggcaca  tgagaccggg ctgaatgcta gcggcaattc catcattcac tacacaaatg ttaattatta  caaggatgcc gcatccaact cagccaatcg gcaggatttc actcaagacc cgggcaagtt  cacagaacca gtaaaagata tcatgattaa atcactacca gctctcaact cccccacagt  agaggagtgc ggatacagtg acagggcgag atcaatcaca |
| 1.48 | 569 | 571 | NHS | ttaaaacagc ctgtgggttg tacccaccca cagggcccac tgggcgctag cactctggta  ttgcggtacc tttgtgcgcc tgttttaccc accctccccc aacgtaactt agaagcctga  catatacggt cacaagccag cccagtacgc caactgggta ccgaccaagc acttctgtta  ccccggactg agtatcaata agctgccaaa gcggctgaag gagaaaacgt tcgttacccg  gccaattact tcgagaaacc cagtaacacc atgaaagttg cgtagcgttt cattcagcac  aatcccagtg tagatcaggt cgatgagtca ccgcattccc cacaggcgac tgtggcggtg  gctgcgttgg cggcctgccc atggggcaaa cccatgggac gcttcaatac tgacatggtg  cgaagagtct attgagctaa ttggtagtcc tccggcccct gaatgcggct aatcctaact  gcggagcagg cactcgcaga ccagcgagca gcttgtcgta atgggcaact ccgcagcgga  accgactact ttgggtgtcc gtgtttcct  tattcctata ctggctgctt atggtgacaa  ttgagagatt gttaccatat agctattgga ttggccatcc ggtgaccaat agagctatta  tatatctctt tgttgggttt ataccactta gcttgaaaga ggttaaaaca ttacaattca  ttgttaagtt gaatacagca aaatgggagc tcaagtatca acgcaaaaga ctggggcaca  tgagaccggg ctgaatgcta gcggcaattc catcattcac tacacaaatg ttaattatta  caaggatgcc gcatccaact cagccaatcg gcaggatttc actcaagacc cgggcaagtt  cacagaacca gtaaaagata tcatgattaa atcactacca gctctcaact cccccacagt  agaggagtgc ggatacagtg acagggcgag atcaatcaca |
| 1.49 | 688 | 650 | HS_B_ | ttaaaacagc ctgtgggttg tacccaccca cagggcccac tgggcgctag cactctggta  ttgcggtacc tttgtgcgcc tgttttaccc accctccccc aacgtaactt agaagcctga  catatacggt cacaagccag cccagtacgc caactgggta ccgaccaagc acttctgtta  ccccggactg agtatcaata agctgccaaa gcggctgaag gagaaaacgt tcgttacccg  gccaattact tcgagaaacc cagtaacacc atgaaagttg cgtagcgttt cattcagcac  aatcccagtg tagatcaggt cgatgagtca ccgcattccc cacaggcgac tgtggcggtg  gctgcgttgg cggcctgccc atggggcaaa cccatgggac gcttcaatac tgacatggtg  cgaagagtct attgagctaa ttggtagtcc tccggcccct gaatgcggct aatcctaact  gcggagcagg cactcgcaga ccagcgagca gcttgtcgta atgggcaact ccgcagcgga  accgactact ttgggtgtcc gtgtttccta tttcctttac actggctgct tatggtgaca  attgagaaat tgttaccata tagctattgg agtggccatc cggtgactaa cagagcaata  atatatttgt ttgttggctt tgtgccac  t agagctatta  tatatctctt tgttgggttt ataccactta gcttgaaaga ggttaaaaca ttacaattca  ttgttaagtt gaatacagca aaatgggagc tcaagtatca acgcaaaaga ctggggcaca  tgagaccggg ctgaatgcta gcggcaattc catcattcac tacacaaatg ttaattatta  caaggatgcc gcatccaact cagccaatcg gcaggatttc actcaagacc cgggcaagtt  cacagaacca gtaaaagata tcatgattaa atcactacca gctctcaact cccccacagt  agaggagtgc ggatacagtg acagggcgag atcaatcaca |
| 1.55 | 127 | 51 | HS_A_ | ttaaaacagc ctgtgggttg tacccaccca cagggcccac tgggcgctag cactctggta  ttgcggtacc tttgtgcgcc tgttttaccc accctccccc aacgtaactt agaagcctga  catatac  cactctggta  tcacggtacc tttgtgcgcc tgttttatac cccctccccc aactgtaact tagaagtaac  acacaccgat caacagtcag cgtggcacac cagccacgtt ttgatcaagc acttctgtta  ccccggactg agtatcaata gactgctcac gcggttgaag gagaaagcgt tcgttatccg  gccaactact tcgaaaaacc tagtaacacc gtggaagttg cagagtgttt cgctcagcac  taccccagtg tagatcaggt cgatgagtca ccgcattccc cacgggcgac cgtggcggtg  gctgcgttgg cggcctgccc atggggaaac ccatgggacg ctctaataca gacatggtgc  gaagagtcta ttgagctagt tggtagtcct ccggcccctg aatgcggcta atcctaactg  cggagcacac accctcaagc cagagggcag tgtgtcgtaa cgggcaactc tgcagcggaa  ccgactactt tgggtgtccg tgtttcattt tattcctata ctggctgctt atggtgacaa  ttgagagatt gttaccatat agctattgga ttggccatcc ggtgaccaat agagctatta  tatatctctt tgttgggttt ataccactta gcttgaaaga ggttaaaaca ttacaattca  ttgttaagtt gaatacagca aaatgggagc tcaagtatca acgcaaaaga ctggggcaca  tgagaccggg ctgaatgcta gcggcaattc catcattcac tacacaaatg ttaattatta  caaggatgcc gcatccaact cagccaatcg gcaggatttc actcaagacc cgggcaagtt  cacagaacca gtaaaagata tcatgattaa atcactacca gctctcaact cccccacagt  agaggagtgc ggatacagtg acagggcgag atcaatcaca |
| 1.56 | 419 | 413 | NHS | ttaaaacagc ctgtgggttg tacccaccca cagggcccac tgggcgctag cactctggta  ttgcggtacc tttgtgcgcc tgttttaccc accctccccc aacgtaactt agaagcctga  catatacggt cacaagccag cccagtacgc caactgggta ccgaccaagc acttctgtta  ccccggactg agtatcaata agctgccaaa gcggctgaag gagaaaacgt tcgttacccg  gccaattact tcgagaaacc cagtaacacc atgaaagttg cgtagcgttt cattcagcac  aatcccagtg tagatcaggt cgatgagtca ccgcattccc cacaggcgac tgtggcggtg  gctgcgttgg cggcctgccc atggggcaaa cccatgggac gcttcaatac tgacatggt  catggtgc  gaagagtcta ttgagctagt tggtagtcct ccggcccctg aatgcggcta atcctaactg  cggagcacac accctcaagc cagagggcag tgtgtcgtaa cgggcaactc tgcagcggaa  ccgactactt tgggtgtccg tgtttcattt tattcctata ctggctgctt atggtgacaa  ttgagagatt gttaccatat agctattgga ttggccatcc ggtgaccaat agagctatta  tatatctctt tgttgggttt ataccactta gcttgaaaga ggttaaaaca ttacaattca  ttgttaagtt gaatacagca aaatgggagc tcaagtatca acgcaaaaga ctggggcaca  tgagaccggg ctgaatgcta gcggcaattc catcattcac tacacaaatg ttaattatta  caaggatgcc gcatccaact cagccaatcg gcaggatttc actcaagacc cgggcaagtt  cacagaacca gtaaaagata tcatgattaa atcactacca gctctcaact cccccacagt  agaggagtgc ggatacagtg acagggcgag atcaatcaca |
| 1.62 | 198 | 51 | HS_A_ | ttaaaacagc ctgtgggttg tacccaccca cagggcccac tgggcgctag cactctggta  ttgcggtacc tttgtgcgcc tgttttaccc accctccccc aacgtaactt agaagcctga  catatacggt cacaagccag cccagtacgc caactgggta ccgaccaagc acttctgtta  ccccggactg agtatcaa  cactctggta  tcacggtacc tttgtgcgcc tgttttatac cccctccccc aactgtaact tagaagtaac  acacaccgat caacagtcag cgtggcacac cagccacgtt ttgatcaagc acttctgtta  ccccggactg agtatcaata gactgctcac gcggttgaag gagaaagcgt tcgttatccg  gccaactact tcgaaaaacc tagtaacacc gtggaagttg cagagtgttt cgctcagcac  taccccagtg tagatcaggt cgatgagtca ccgcattccc cacgggcgac cgtggcggtg  gctgcgttgg cggcctgccc atggggaaac ccatgggacg ctctaataca gacatggtgc  gaagagtcta ttgagctagt tggtagtcct ccggcccctg aatgcggcta atcctaactg  cggagcacac accctcaagc cagagggcag tgtgtcgtaa cgggcaactc tgcagcggaa  ccgactactt tgggtgtccg tgtttcattt tattcctata ctggctgctt atggtgacaa  ttgagagatt gttaccatat agctattgga ttggccatcc ggtgaccaat agagctatta  tatatctctt tgttgggttt ataccactta gcttgaaaga ggttaaaaca ttacaattca  ttgttaagtt gaatacagca aaatgggagc tcaagtatca acgcaaaaga ctggggcaca  tgagaccggg ctgaatgcta gcggcaattc catcattcac tacacaaatg ttaattatta  caaggatgcc gcatccaact cagccaatcg gcaggatttc actcaagacc cgggcaagtt  cacagaacca gtaaaagata tcatgattaa atcactacca gctctcaact cccccacagt  agaggagtgc ggatacagtg acagggcgag atcaatcaca |
| 1.66 | 134 | 51 | HS_A_ | ttaaaacagc ctgtgggttg tacccaccca cagggcccac tgggcgctag cactctggta  ttgcggtacc tttgtgcgcc tgttttaccc accctccccc aacgtaactt agaagcctga  catatacggt caca  cactctggta  tcacggtacc tttgtgcgcc tgttttatac cccctccccc aactgtaact tagaagtaac  acacaccgat caacagtcag cgtggcacac cagccacgtt ttgatcaagc acttctgtta  ccccggactg agtatcaata gactgctcac gcggttgaag gagaaagcgt tcgttatccg  gccaactact tcgaaaaacc tagtaacacc gtggaagttg cagagtgttt cgctcagcac  taccccagtg tagatcaggt cgatgagtca ccgcattccc cacgggcgac cgtggcggtg  gctgcgttgg cggcctgccc atggggaaac ccatgggacg ctctaataca gacatggtgc  gaagagtcta ttgagctagt tggtagtcct ccggcccctg aatgcggcta atcctaactg  cggagcacac accctcaagc cagagggcag tgtgtcgtaa cgggcaactc tgcagcggaa  ccgactactt tgggtgtccg tgtttcattt tattcctata ctggctgctt atggtgacaa  ttgagagatt gttaccatat agctattgga ttggccatcc ggtgaccaat agagctatta  tatatctctt tgttgggttt ataccactta gcttgaaaga ggttaaaaca ttacaattca  ttgttaagtt gaatacagca aaatgggagc tcaagtatca acgcaaaaga ctggggcaca  tgagaccggg ctgaatgcta gcggcaattc catcattcac tacacaaatg ttaattatta  caaggatgcc gcatccaact cagccaatcg gcaggatttc actcaagacc cgggcaagtt  cacagaacca gtaaaagata tcatgattaa atcactacca gctctcaact cccccacagt  agaggagtgc ggatacagtg acagggcgag atcaatcaca |
| 1.69 | 134 | 51 | HS_A_ | ttaaaacagc ctgtgggttg tacccaccca cagggcccac tgggcgctag cactctggta  ttgcggtacc tttgtgcgcc tgttttaccc accctccccc aacgtaactt agaagcctga  catatacggt caca  cactctggta  tcacggtacc tttgtgcgcc tgttttatac cccctccccc aactgtaact tagaagtaac  acacaccgat caacagtcag cgtggcacac cagccacgtt ttgatcaagc acttctgtta  ccccggactg agtatcaata gactgctcac gcggttgaag gagaaagcgt tcgttatccg  gccaactact tcgaaaaacc tagtaacacc gtggaagttg cagagtgttt cgctcagcac  taccccagtg tagatcaggt cgatgagtca ccgcattccc cacgggcgac cgtggcggtg  gctgcgttgg cggcctgccc atggggaaac ccatgggacg ctctaataca gacatggtgc  gaagagtcta ttgagctagt tggtagtcct ccggcccctg aatgcggcta atcctaactg  cggagcacac accctcaagc cagagggcag tgtgtcgtaa cgggcaactc tgcagcggaa  ccgactactt tgggtgtccg tgtttcattt tattcctata ctggctgctt atggtgacaa  ttgagagatt gttaccatat agctattgga ttggccatcc ggtgaccaat agagctatta  tatatctctt tgttgggttt ataccactta gcttgaaaga ggttaaaaca ttacaattca  ttgttaagtt gaatacagca aaatgggagc tcaagtatca acgcaaaaga ctggggcaca  tgagaccggg ctgaatgcta gcggcaattc catcattcac tacacaaatg ttaattatta  caaggatgcc gcatccaact cagccaatcg gcaggatttc actcaagacc cgggcaagtt  cacagaacca gtaaaagata tcatgattaa atcactacca gctctcaact cccccacagt  agaggagtgc ggatacagtg acagggcgag atcaatcaca |
| 2.13 | 90 | 87 | HS_A_ | ttaaaacagc ctgtgggttg tacccaccca cagggcccac tgggcgctag cactctggta  ttgcggtacc tttgtgcgcc tgttttaccc  atac cccctccccc aactgtaact tagaagtaac  acacaccgat caacagtcag cgtggcacac cagccacgtt ttgatcaagc acttctgtta  ccccggactg agtatcaata gactgctcac gcggttgaag gagaaagcgt tcgttatccg  gccaactact tcgaaaaacc tagtaacacc gtggaagttg cagagtgttt cgctcagcac  taccccagtg tagatcaggt cgatgagtca ccgcattccc cacgggcgac cgtggcggtg  gctgcgttgg cggcctgccc atggggaaac ccatgggacg ctctaataca gacatggtgc  gaagagtcta ttgagctagt tggtagtcct ccggcccctg aatgcggcta atcctaactg  cggagcacac accctcaagc cagagggcag tgtgtcgtaa cgggcaactc tgcagcggaa  ccgactactt tgggtgtccg tgtttcattt tattcctata ctggctgctt atggtgacaa  ttgagagatt gttaccatat agctattgga ttggccatcc ggtgaccaat agagctatta  tatatctctt tgttgggttt ataccactta gcttgaaaga ggttaaaaca ttacaattca  ttgttaagtt gaatacagca aaatgggagc tcaagtatca acgcaaaaga ctggggcaca  tgagaccggg ctgaatgcta gcggcaattc catcattcac tacacaaatg ttaattatta  caaggatgcc gcatccaact cagccaatcg gcaggatttc actcaagacc cgggcaagtt  cacagaacca gtaaaagata tcatgattaa atcactacca gctctcaact cccccacagt  agaggagtgc ggatacagtg acagggcgag atcaatcaca |
| 2.14 | 697 | 683 | HS_B_ | ttaaaacagc ctgtgggttg tacccaccca cagggcccac tgggcgctag cactctggta  ttgcggtacc tttgtgcgcc tgttttaccc accctccccc aacgtaactt agaagcctga  catatacggt cacaagccag cccagtacgc caactgggta ccgaccaagc acttctgtta  ccccggactg agtatcaata agctgccaaa gcggctgaag gagaaaacgt tcgttacccg  gccaattact tcgagaaacc cagtaacacc atgaaagttg cgtagcgttt cattcagcac  aatcccagtg tagatcaggt cgatgagtca ccgcattccc cacaggcgac tgtggcggtg  gctgcgttgg cggcctgccc atggggcaaa cccatgggac gcttcaatac tgacatggtg  cgaagagtct attgagctaa ttggtagtcc tccggcccct gaatgcggct aatcctaact  gcggagcagg cactcgcaga ccagcgagca gcttgtcgta atgggcaact ccgcagcgga  accgactact ttgggtgtcc gtgtttccta tttcctttac actggctgct tatggtgaca  attgagaaat tgttaccata tagctattgg agtggccatc cggtgactaa cagagcaata  atatatttgt ttgttggctt tgtgccactt aatttga  accactta gcttgaaaga ggttaaaaca ttacaattca  ttgttaagtt gaatacagca aaatgggagc tcaagtatca acgcaaaaga ctggggcaca  tgagaccggg ctgaatgcta gcggcaattc catcattcac tacacaaatg ttaattatta  caaggatgcc gcatccaact cagccaatcg gcaggatttc actcaagacc cgggcaagtt  cacagaacca gtaaaagata tcatgattaa atcactacca gctctcaact cccccacagt  agaggagtgc ggatacagtg acagggcgag atcaatcaca |
| 2.16 | 126 | 125 | HS_A_ | ttaaaacagc ctgtgggttg tacccaccca cagggcccac tgggcgctag cactctggta  ttgcggtacc tttgtgcgcc tgttttaccc accctccccc aacgtaactt agaagcctga  catata  accgat caacagtcag cgtggcacac cagccacgtt ttgatcaagc acttctgtta  ccccggactg agtatcaata gactgctcac gcggttgaag gagaaagcgt tcgttatccg  gccaactact tcgaaaaacc tagtaacacc gtggaagttg cagagtgttt cgctcagcac  taccccagtg tagatcaggt cgatgagtca ccgcattccc cacgggcgac cgtggcggtg  gctgcgttgg cggcctgccc atggggaaac ccatgggacg ctctaataca gacatggtgc  gaagagtcta ttgagctagt tggtagtcct ccggcccctg aatgcggcta atcctaactg  cggagcacac accctcaagc cagagggcag tgtgtcgtaa cgggcaactc tgcagcggaa  ccgactactt tgggtgtccg tgtttcattt tattcctata ctggctgctt atggtgacaa  ttgagagatt gttaccatat agctattgga ttggccatcc ggtgaccaat agagctatta  tatatctctt tgttgggttt ataccactta gcttgaaaga ggttaaaaca ttacaattca  ttgttaagtt gaatacagca aaatgggagc tcaagtatca acgcaaaaga ctggggcaca  tgagaccggg ctgaatgcta gcggcaattc catcattcac tacacaaatg ttaattatta  caaggatgcc gcatccaact cagccaatcg gcaggatttc actcaagacc cgggcaagtt  cacagaacca gtaaaagata tcatgattaa atcactacca gctctcaact cccccacagt  agaggagtgc ggatacagtg acagggcgag atcaatcaca |
| 2.18 | 90 | 87 | HS_A_ | ttaaaacagc ctgtgggttg tacccaccca cagggcccac tgggcgctag cactctggta  ttgcggtacc tttgtgcgcc tgttttaccc  atac cccctccccc aactgtaact tagaagtaac  acacaccgat caacagtcag cgtggcacac cagccacgtt ttgatcaagc acttctgtta  ccccggactg agtatcaata gactgctcac gcggttgaag gagaaagcgt tcgttatccg  gccaactact tcgaaaaacc tagtaacacc gtggaagttg cagagtgttt cgctcagcac  taccccagtg tagatcaggt cgatgagtca ccgcattccc cacgggcgac cgtggcggtg  gctgcgttgg cggcctgccc atggggaaac ccatgggacg ctctaataca gacatggtgc  gaagagtcta ttgagctagt tggtagtcct ccggcccctg aatgcggcta atcctaactg  cggagcacac accctcaagc cagagggcag tgtgtcgtaa cgggcaactc tgcagcggaa  ccgactactt tgggtgtccg tgtttcattt tattcctata ctggctgctt atggtgacaa  ttgagagatt gttaccatat agctattgga ttggccatcc ggtgaccaat agagctatta  tatatctctt tgttgggttt ataccactta gcttgaaaga ggttaaaaca ttacaattca  ttgttaagtt gaatacagca aaatgggagc tcaagtatca acgcaaaaga ctggggcaca  tgagaccggg ctgaatgcta gcggcaattc catcattcac tacacaaatg ttaattatta  caaggatgcc gcatccaact cagccaatcg gcaggatttc actcaagacc cgggcaagtt  cacagaacca gtaaaagata tcatgattaa atcactacca gctctcaact cccccacagt  agaggagtgc ggatacagtg acagggcgag atcaatcaca |
| 2.30 | 173 | 104 | HS_A_ | ttaaaacagc ctgtgggttg tacccaccca cagggcccac tgggcgctag cactctggta  ttgcggtacc tttgtgcgcc tgttttaccc accctccccc aacgtaactt agaagcctga  catatacggt cacaagccag cccagtacgc caactgggta ccgaccaagc act  tgtaact tagaagtaac  acacaccgat caacagtcag cgtggcacac cagccacgtt ttgatcaagc acttctgtta  ccccggactg agtatcaata gactgctcac gcggttgaag gagaaagcgt tcgttatccg  gccaactact tcgaaaaacc tagtaacacc gtggaagttg cagagtgttt cgctcagcac  taccccagtg tagatcaggt cgatgagtca ccgcattccc cacgggcgac cgtggcggtg  gctgcgttgg cggcctgccc atggggaaac ccatgggacg ctctaataca gacatggtgc  gaagagtcta ttgagctagt tggtagtcct ccggcccctg aatgcggcta atcctaactg  cggagcacac accctcaagc cagagggcag tgtgtcgtaa cgggcaactc tgcagcggaa  ccgactactt tgggtgtccg tgtttcattt tattcctata ctggctgctt atggtgacaa  ttgagagatt gttaccatat agctattgga ttggccatcc ggtgaccaat agagctatta  tatatctctt tgttgggttt ataccactta gcttgaaaga ggttaaaaca ttacaattca  ttgttaagtt gaatacagca aaatgggagc tcaagtatca acgcaaaaga ctggggcaca  tgagaccggg ctgaatgcta gcggcaattc catcattcac tacacaaatg ttaattatta  caaggatgcc gcatccaact cagccaatcg gcaggatttc actcaagacc cgggcaagtt  cacagaacca gtaaaagata tcatgattaa atcactacca gctctcaact cccccacagt  agaggagtgc ggatacagtg acagggcgag atcaatcaca |
| 3.37 | 185 | 50 | HS_A_ | ttaaaacagc ctgtgggttg tacccaccca cagggcccac tgggcgctag cactctggta  ttgcggtacc tttgtgcgcc tgttttaccc accctccccc aacgtaactt agaagcctga  catatacggt cacaagccag cccagtacgc caactgggta ccgaccaagc acttctgtta  ccccg  g cactctggta  tcacggtacc tttgtgcgcc tgttttatac cccctccccc aactgtaact tagaagtaac  acacaccgat caacagtcag cgtggcacac cagccacgtt ttgatcaagc acttctgtta  ccccggactg agtatcaata gactgctcac gcggttgaag gagaaagcgt tcgttatccg  gccaactact tcgaaaaacc tagtaacacc gtggaagttg cagagtgttt cgctcagcac  taccccagtg tagatcaggt cgatgagtca ccgcattccc cacgggcgac cgtggcggtg  gctgcgttgg cggcctgccc atggggaaac ccatgggacg ctctaataca gacatggtgc  gaagagtcta ttgagctagt tggtagtcct ccggcccctg aatgcggcta atcctaactg  cggagcacac accctcaagc cagagggcag tgtgtcgtaa cgggcaactc tgcagcggaa  ccgactactt tgggtgtccg tgtttcattt tattcctata ctggctgctt atggtgacaa  ttgagagatt gttaccatat agctattgga ttggccatcc ggtgaccaat agagctatta  tatatctctt tgttgggttt ataccactta gcttgaaaga ggttaaaaca ttacaattca  ttgttaagtt gaatacagca aaatgggagc tcaagtatca acgcaaaaga ctggggcaca  tgagaccggg ctgaatgcta gcggcaattc catcattcac tacacaaatg ttaattatta  caaggatgcc gcatccaact cagccaatcg gcaggatttc actcaagacc cgggcaagtt  cacagaacca gtaaaagata tcatgattaa atcactacca gctctcaact cccccacagt  agaggagtgc ggatacagtg acagggcgag atcaatcaca |
| 3.39 | 175 | 113 | HS_A_ | ttaaaacagc ctgtgggttg tacccaccca cagggcccac tgggcgctag cactctggta  ttgcggtacc tttgtgcgcc tgttttaccc accctccccc aacgtaactt agaagcctga  catatacggt cacaagccag cccagtacgc caactgggta ccgaccaagc acttc  gaagtaac  acacaccgat caacagtcag cgtggcacac cagccacgtt ttgatcaagc acttctgtta  ccccggactg agtatcaata gactgctcac gcggttgaag gagaaagcgt tcgttatccg  gccaactact tcgaaaaacc tagtaacacc gtggaagttg cagagtgttt cgctcagcac  taccccagtg tagatcaggt cgatgagtca ccgcattccc cacgggcgac cgtggcggtg  gctgcgttgg cggcctgccc atggggaaac ccatgggacg ctctaataca gacatggtgc  gaagagtcta ttgagctagt tggtagtcct ccggcccctg aatgcggcta atcctaactg  cggagcacac accctcaagc cagagggcag tgtgtcgtaa cgggcaactc tgcagcggaa  ccgactactt tgggtgtccg tgtttcattt tattcctata ctggctgctt atggtgacaa  ttgagagatt gttaccatat agctattgga ttggccatcc ggtgaccaat agagctatta  tatatctctt tgttgggttt ataccactta gcttgaaaga ggttaaaaca ttacaattca  ttgttaagtt gaatacagca aaatgggagc tcaagtatca acgcaaaaga ctggggcaca  tgagaccggg ctgaatgcta gcggcaattc catcattcac tacacaaatg ttaattatta  caaggatgcc gcatccaact cagccaatcg gcaggatttc actcaagacc cgggcaagtt  cacagaacca gtaaaagata tcatgattaa atcactacca gctctcaact cccccacagt  agaggagtgc ggatacagtg acagggcgag atcaatcaca |
| 3.43 | 638 | 630 | HS_B_ | ttaaaacagc ctgtgggttg tacccaccca cagggcccac tgggcgctag cactctggta  ttgcggtacc tttgtgcgcc tgttttaccc accctccccc aacgtaactt agaagcctga  catatacggt cacaagccag cccagtacgc caactgggta ccgaccaagc acttctgtta  ccccggactg agtatcaata agctgccaaa gcggctgaag gagaaaacgt tcgttacccg  gccaattact tcgagaaacc cagtaacacc atgaaagttg cgtagcgttt cattcagcac  aatcccagtg tagatcaggt cgatgagtca ccgcattccc cacaggcgac tgtggcggtg  gctgcgttgg cggcctgccc atggggcaaa cccatgggac gcttcaatac tgacatggtg  cgaagagtct attgagctaa ttggtagtcc tccggcccct gaatgcggct aatcctaact  gcggagcagg cactcgcaga ccagcgagca gcttgtcgta atgggcaact ccgcagcgga  accgactact ttgggtgtcc gtgtttccta tttcctttac actggctgct tatggtgaca  attgagaaat tgttaccata tagctattgg agtggcca  a ttggccatcc ggtgaccaat agagctatta  tatatctctt tgttgggttt ataccactta gcttgaaaga ggttaaaaca ttacaattca  ttgttaagtt gaatacagca aaatgggagc tcaagtatca acgcaaaaga ctggggcaca  tgagaccggg ctgaatgcta gcggcaattc catcattcac tacacaaatg ttaattatta  caaggatgcc gcatccaact cagccaatcg gcaggatttc actcaagacc cgggcaagtt  cacagaacca gtaaaagata tcatgattaa atcactacca gctctcaact cccccacagt  agaggagtgc ggatacagtg acagggcgag atcaatcaca |
| 3.44 | 134 | 51 | HS_A_ | ttaaaacagc ctgtgggttg tacccaccca cagggcccac tgggcgctag cactctggta  ttgcggtacc tttgtgcgcc tgttttaccc accctccccc aacgtaactt agaagcctga  catatacggt caca  cactctggta  tcacggtacc tttgtgcgcc tgttttatac cccctccccc aactgtaact tagaagtaac  acacaccgat caacagtcag cgtggcacac cagccacgtt ttgatcaagc acttctgtta  ccccggactg agtatcaata gactgctcac gcggttgaag gagaaagcgt tcgttatccg  gccaactact tcgaaaaacc tagtaacacc gtggaagttg cagagtgttt cgctcagcac  taccccagtg tagatcaggt cgatgagtca ccgcattccc cacgggcgac cgtggcggtg  gctgcgttgg cggcctgccc atggggaaac ccatgggacg ctctaataca gacatggtgc  gaagagtcta ttgagctagt tggtagtcct ccggcccctg aatgcggcta atcctaactg  cggagcacac accctcaagc cagagggcag tgtgtcgtaa cgggcaactc tgcagcggaa  ccgactactt tgggtgtccg tgtttcattt tattcctata ctggctgctt atggtgacaa  ttgagagatt gttaccatat agctattgga ttggccatcc ggtgaccaat agagctatta  tatatctctt tgttgggttt ataccactta gcttgaaaga ggttaaaaca ttacaattca  ttgttaagtt gaatacagca aaatgggagc tcaagtatca acgcaaaaga ctggggcaca  tgagaccggg ctgaatgcta gcggcaattc catcattcac tacacaaatg ttaattatta  caaggatgcc gcatccaact cagccaatcg gcaggatttc actcaagacc cgggcaagtt  cacagaacca gtaaaagata tcatgattaa atcactacca gctctcaact cccccacagt  agaggagtgc ggatacagtg acagggcgag atcaatcaca |
