# Supplementary material for: EV-B 3D polymerase remodels viral populations through 5′UTR recombination to subvert cardiac antiviral innate immunity
Source: PLoS Pathog. 2026 Jul 24;22(7):e1014441. doi: 10.1371/journal.ppat.1014441 (PMC13426973; doi:10.1371/journal.ppat.1014441)

Figure 7 panel E: iBright CL1500 Image Analysis Report

P_TBK1_HCM

Date:	24 September 2025 12:57:25PM
Mode:	Chemi Blots
Notes:	
Model:	CL1500
Instrument name:	2462521100009
Serial No:	2462521100009
Firmware version:	1.8.0
iBA version:	5.5.0
Image size:	352px X 282px
Image area:	112.7mm X 90.16mm
Optical Zoom:	2x
Digital Zoom:	1.2x
Focus level:	420
Resolution:	8 x 8
Exposure time:	3029 ms
Exposure mode:	Normal

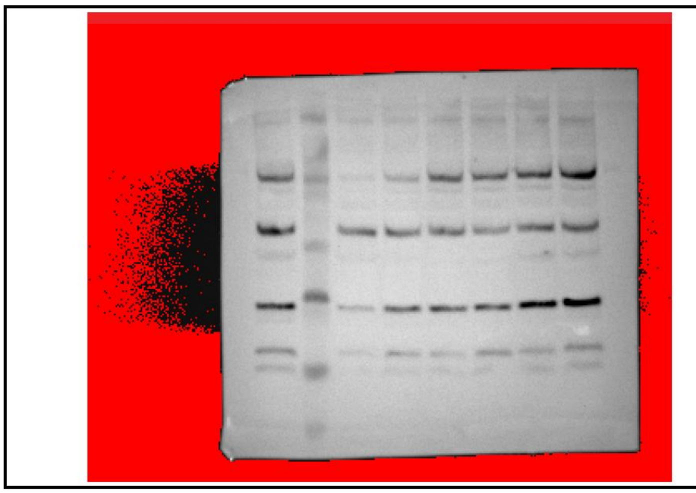

TBK1_HCM

Date:	24 September 2025 05:07:31PM
Mode:	Chemi Blots
Notes:	
Model:	CL1500
Instrument name:	2462521100009
Serial No:	2462521100009
Firmware version:	1.8.0
iBA version:	5.5.0
Image size:	423px X 338px
Image area:	125.22mm X 100.18mm
Optical Zoom:	1.8x
Digital Zoom:	1x
Focus level:	375
Resolution:	8 x 8
Exposure time:	2000 ms
Exposure mode:	Normal

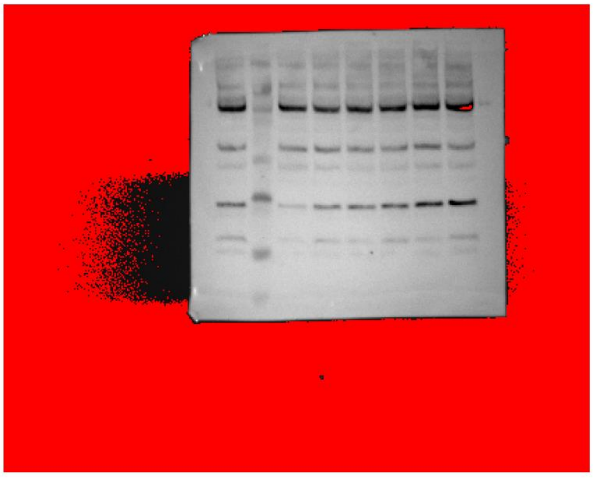

BACTIN_HCM

Date:	26 September 2025 02:40:15PM
Mode:	Chemi Blots
Notes:	
Model:	CL1500
Instrument name:	2462521100009
Serial No:	2462521100009
Firmware version:	1.8.0
iBA version:	5.5.0
Image size:	385px X 307px
Image area:	112.7mm X 90.16mm
Optical Zoom:	2x
Digital Zoom:	1.1x
Focus level:	420
Resolution:	8 x 8
Exposure time:	4500 ms
Exposure mode:	Normal

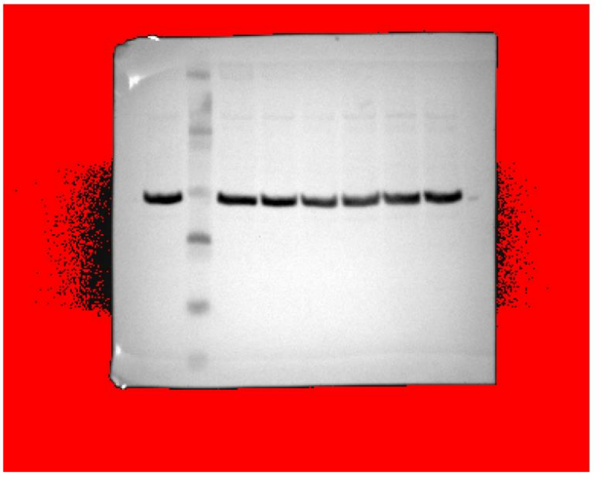

Figure 7 panel E: annotation of the loading order

TBK1-phosphorylated revelation

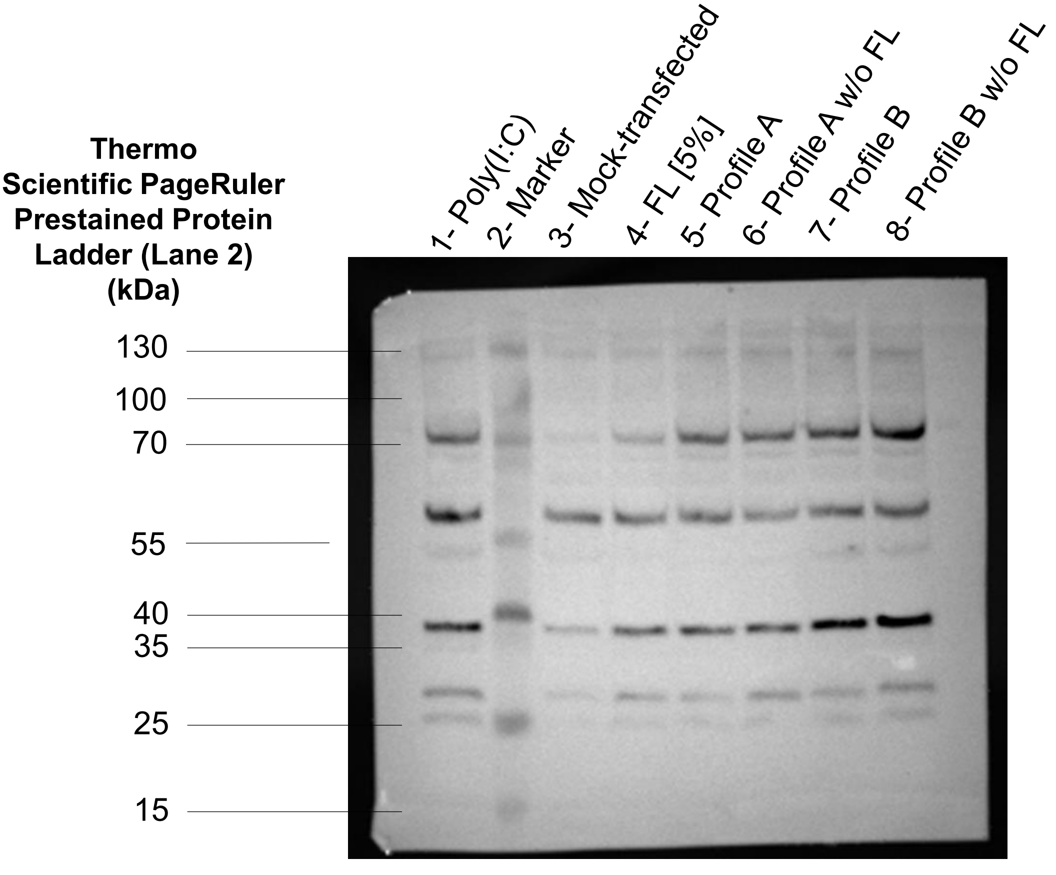

Figure 7 panel E: annotation of the loading order

TBK1-phosphorylated revelation

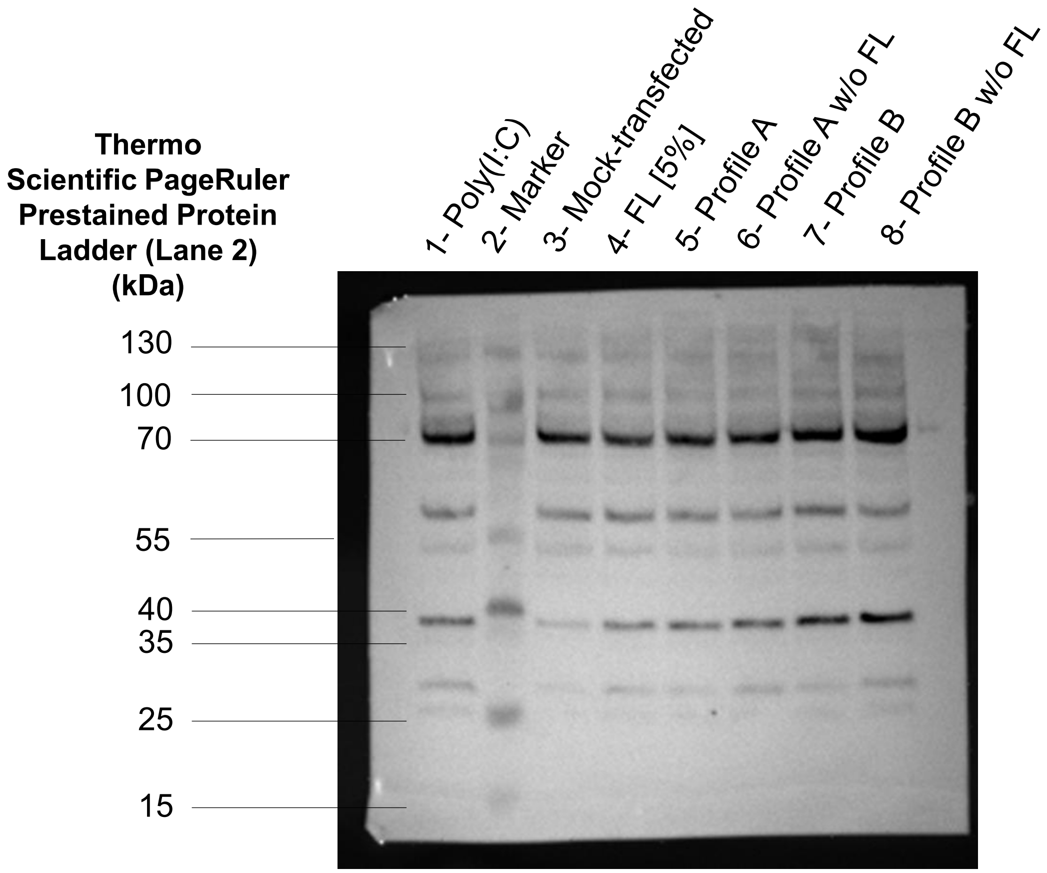

Figure 7 panel E: annotation of the loading order

TBK1-phosphorylated revelation

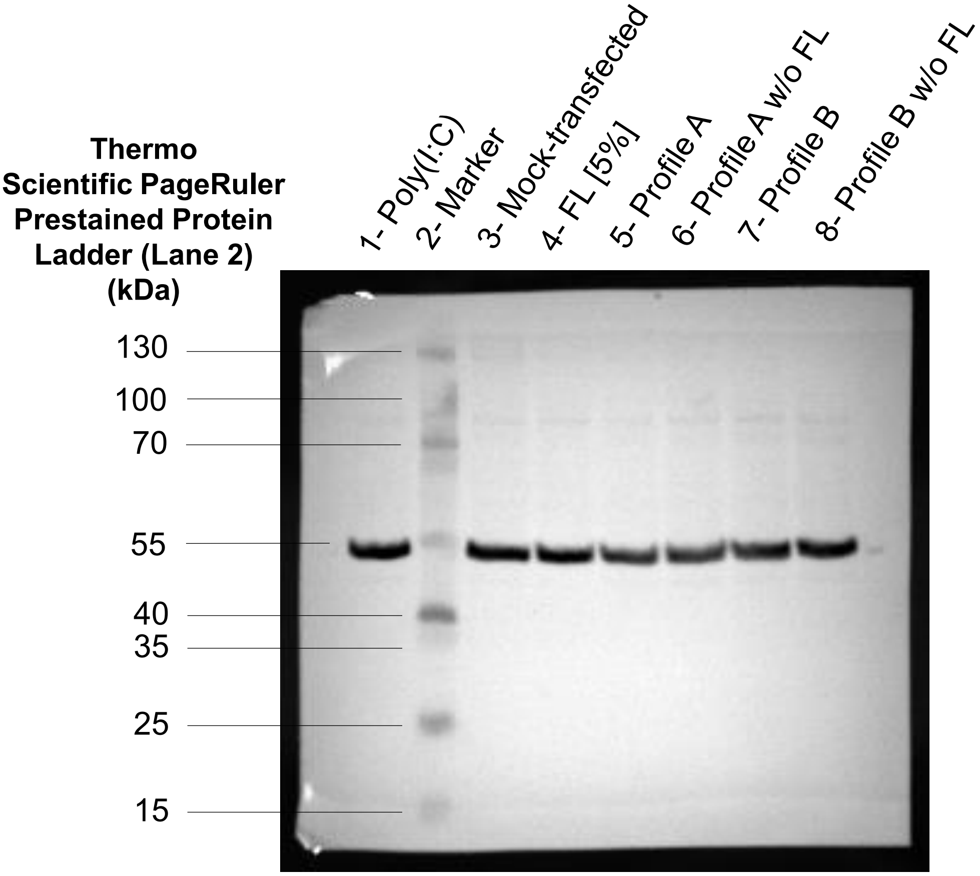

Supplement: S1 Raw Image — The loading order was as follows: lane 1, high molecular weight (HMW) poly(I:C) used as a positive control; lane 2, molecular weight marker (PageRuler Prestained Protein Ladder, Thermo Fisher Scientific); lane 3, mock-transfected; lane 4, FL (5%); lane 5, Profile A; lane 6, Profile A without FL; lane 7, Profile B; lane 8, Profile B without FL. (PDF) [file ppat.1014441.s006.pdf]
